# Global Burden of Iodine Deficiency: Insights and Projections to 2050 Using XGBoost and SHAP

**DOI:** 10.1016/j.advnut.2025.100384

**Published:** 2025-02-04

**Authors:** Dan Liang, Li Wang, Panpan Zhong, Jiuxiu Lin, Leyan Chen, Qifang Chen, Shuang Liu, Zhen Luo, Changwen Ke, Yingsi Lai

**Affiliations:** 1 Department of Immunology and Microbiology, College of Life Science and Technology, Jinan University, Guangzhou, China; 2 Department of Medical Statistics, School of Public Health, Sun Yat-sen University, Guangzhou, China; 3 Department of Microbiology and Immunology, Basic Medicine College, Jinan University, Guangzhou, China; 4 International Institute for Translational Chinese Medicine, School of Chinese Materia Medica, Guangzhou University of Chinese Medicine, Guangzhou, China; 5 BSL-3 Laboratory (Guangdong), Guangdong Provincial Key Laboratory of Tropical Disease Research, School of Public Health, Southern Medical University, Guangzhou, China; 6 Guangdong Provincial Key Laboratory for Emergency Detection and Research on Pathogen of Emerging Infectious Disease, Guangdong Provincial Center for Disease Control and Prevention, Guangdong Workstation for Emerging Infectious Disease Control and Prevention, Guangzhou, Guangdong, China

**Keywords:** iodine deficiency, Global Burden of Disease, SHAP, XGBoost, epidemiology, forecasting

## Abstract

Iodine deficiency (ID) poses a significant global public health challenge. This study aimed to analyze trends from 1990 to 2021 and project future patterns ≤2050 using the extreme gradient boosting (XGBoost) model, with Shapley additive explanations (SHAP), to identify key factors and inform public health strategies. Data on ID from the Global Burden of Disease (GBD) 2021 study were used to model and predict its burden ≤2050 using XGBoost, with SHAP enhancing model interpretability. In 1990, global incident cases of ID were 7.51 million (age-standardized incidence rate [ASIR]: 126.11/100,000), rising to 8.08 million by 2021 (ASIR: 105.99/100,000, a 15.96% decrease), and projected to reach 8.48 million by 2050 (ASIR: 108.20/100,000). Prevalent cases increased from 146.42 million in 1990 (age-standardized prevalence rate [ASPR]: 2801.80/100,000) to 180.81 million in 2021 (ASPR: 2213.98/100,000, a 20.98% decrease), with 194.51 million expected by 2050 (ASPR: 1900.01/100,000). Disability-adjusted life years (DALYs) dropped from 2.46 million in 1990 (age-standardized disability-adjusted life year rate [ASDR]: 46.19/100,000) to 2.25 million in 2021 (ASDR: 27.67/100,000, a 40.10% decrease) but are projected to rise slightly to 2.51 million by 2050 (ASDR: 25.51/100,000). SHAP analysis identified iodized salt coverage as a key factor, with higher coverage levels associated with reduced ID burden in most countries. Women and people aged 10–30 y had higher incidence rates, although prevalence and DALYs peaked among those aged 20–45 y. Central and Eastern Sub-Saharan Africa and South Asia will continue to bear the highest burden through 2050. The XGBoost+SHAP model identified age, sex, and iodized salt coverage as key factors, with women and younger populations being high-risk groups. Strengthening iodization programs, improving health care access, targeted education, and consistent monitoring of vulnerable populations are essential to mitigate future risks and improve health outcomes.

**Statements of significance**

This study breaks new ground by applying the XGBoost model paired with SHAP analysis to not only precisely project iodine deficiency trends from 1990 to 2050 but also comprehensively identify age, sex, and iodized salt coverage as key factors that have not been comprehensively explored in this way in previous research, thus offering novel insights crucial for formulating targeted public health strategies in the field of ID research.

## Introduction

Iodine is an essential trace element, critical for numerous bodily functions, particularly the production of thyroid hormones (T4 and T3), which regulate metabolism, growth, and development [1]. Iodine also plays a crucial role in neurodevelopment, especially during fetal and early childhood stages [2]. Beyond its role in thyroid hormone synthesis, iodine exhibits antioxidant properties, reducing oxidative stress and supporting immune function [3,4]. ID is most common in areas with very low dietary iodine, particularly remote inland areas where seafood is not commonly consumed. Severe ID can lead to a range of adverse health outcomes, including thyroid enlargement (goiter), thyroid dysfunction, cognitive impairment, and, in extreme cases, cretinism—a severe and irreversible form of mental retardation [2,5]. ID was a considerable contributor to the global burden of disease. In 2019, the global ASIR of ID was 108.32/100,000, with Asia alone reporting a staggering 127 million cases, 5.47 million new cases, and 1.77 million DALYs attributed to ID [6,7]. These figures highlight the widespread impact of ID, particularly in regions where dietary iodine remains inadequate.

Accurately predicting ID trends is critical for effective public health planning. Regions affected by severe ID often face additional challenges, such as widespread cognitive impairment and reduced economic productivity, which underscore the urgency of implementing evidence-based interventions. Moreover, understanding how ID may evolve over time is essential for identifying vulnerable populations and guiding strategies to mitigate its health and societal impacts. Reliable prediction models are needed to anticipate how ID may evolve over time, especially in regions where dietary changes or public health interventions could alter trends.

Traditional forecasting models, such as age-period-cohort (APC), Bayesian age-period-cohort (BAPC), and autoregressive integrated moving average (ARIMA), have been widely used to model and predict disease trends. APC and BAPC models focus on disentangling the influence of age, period, and cohort effects. Although effective in capturing broad epidemiologic trends, these models face significant challenges when applied to dynamic and nonlinear health data, such as ID. Specifically, BAPC models rely on strong assumptions about the independence of age, period, and cohort effects, which can lead to identifiability issues that require the imposition of additional constraints, such as smoothness constraints [8]. These constraints often make BAPC models less capable of capturing abrupt changes or irregular trends that arise from external factors, such as public health interventions or changes in dietary behavior. For example, iodine fortification programs can lead to abrupt decreases or even reversals in deficiency trends, which are not well-accounted for in these models [9]. Moreover, BAPC models are primarily designed for retrospective analyses and may struggle with forecasting when external policy or environmental changes play a significant role in shaping future trends. Similarly, ARIMA models, commonly used in time series forecasting, perform well in capturing linear trends based on historical data but struggle with nonlinear patterns and complex external influences, such as environmental changes or public health interventions. These limitations make ARIMA less suitable for modeling the dynamic nature of ID, where nonlinear relationships and interactions with external variables play a critical role [10].

Machine learning models, particularly XGBoost have gained attention due to their ability to capture complex, nonlinear relationships more effectively than traditional models. However, a challenge with machine learning models is their “black box” nature, meaning that although they often produce highly accurate predictions, understanding how they make those predictions is not straightforward. This is where SHAP comes into play [11]. SHAP clarifies how machine learning models, like XGBoost, generate predictions by quantifying the impact of each feature. It assigns a value to each feature that shows how much it shifts the prediction, making the model’s decisions easier to interpret and providing actionable insights for decision-makers [12]. In health research, combining XGBoost with SHAP has significantly improved both predictive accuracy and interpretability. XGBoost outperformed logistic regression in predicting myocardial infarction, with SHAP explaining key factors behind predictions, providing a clearer understanding of risk factors in a large cohort study [13]. Similarly, XGBoost and SHAP were used to predict central cervical lymph node metastasis in papillary thyroid carcinoma, identifying critical features such as capsular invasion and radiomics scores to improve clinical decision-making [14]. XGBoost and SHAP have also been applied to global diarrheal disease prediction, highlighting risk factors such as age older than 60 y and inadequate access to safe water, further demonstrating the versatility and utility of these models in health prediction tasks [15].

In this research, we used the GBD 2021 study, which is conducted by the Institute for Health Metrics and Evaluation (IHME). As a comprehensive global initiative, GBD 2021 provides standardized estimates of disease prevalence, incidence, mortality, and DALYs. Drawing from a wide array of data sources, including health surveys, administrative records, and epidemiologic studies from countries worldwide, it offers critical insights into global, regional, and national health trends. These insights make it a key resource for shaping public health policies and interventions [16]. By leveraging GBD 2021, we applied XGBoost combined with SHAP to model and predict ID trends globally, regionally, and nationally from 1990 to 2021 and projected trends from 2022 to 2050. In this study, ID was assessed using the GBD 2021 framework, defining it through clinically significant outcomes such as visible goiter (grade 2) and its associated sequelae. Subclinical ID and mild goiter (grade 1) were excluded from this definition. Although the GBD case definition does not stratify by age or biochemical markers, our analysis results are presented across different age groups to provide detailed insights into population-level trends. The findings from this study aimed to provide actionable insights for reducing the burden of ID through evidence-based public health strategies.

## Methods

### Data source and definitions

The data for this study, which aimed to assess the global burden of ID using key measures such as incidence, prevalence, and DALYs, were sourced from the GBD 2021 Results Tool (https://vizhub.healthdata.org/gbd-results/). Developed by the GBD collaborators, this tool provides comprehensive insights into various health conditions. ID data were extracted for the period 1990–2021, covering 204 countries and territories across 21 GBD regions. The data set is stratified by year, sex, and broad age groups, ranging from younger than 5 y to older than 85 y in 5-y intervals.

ID in this study followed the GBD 2021 framework [17], defining the condition through clinical manifestations, specifically visible goiter (grade 2) and its associated sequelae such as thyroid dysfunction, intellectual disabilities, and heart failure. Subclinical ID and mild goiter (grade 1) were excluded from the case definition, and the GBD framework does not incorporate biochemical indicators such as urinary iodine concentrations. The GBD framework does not explicitly specify whether specific subpopulations, such as pregnant women, are included or excluded. To align with the GBD methodology, our study adopted the same definition and analyzed ID at a general population level.

Population estimates for 1990–2021 were derived by dividing the number of new ID cases by the corresponding incidence rates. Projections for population data from 2022 to 2050 were sourced from IHME [18]. The sociodemographic index (SDI), combining metrics such as income per capita, education levels, and fertility rates, was also obtained from IHME for the years 1990–2021 [19]. The SDI provides a valuable framework for analyzing the impact of socioeconomic conditions on ID, enabling a deeper understanding of health disparities across different regions.

The iodized salt coverage data for 66 countries were sourced from the WHO’s Nutrition Landscape Information System [20]. Iodized salt coverage is defined as the percentage of households consuming adequately iodized salt, which is salt containing ≥15 parts per million. The data span from 1990 to 2021, and these 66 countries were selected because they have data at the national level for ≥3 years, allowing for more reliable imputation of missing values. In contrast, the majority of countries have fewer than 3 y of national-level data, or no data at all, making them unsuitable for this study’s modeling strategy. (A full list of the 66 countries included in the analysis, based on available iodized salt coverage data for ≥3 y, is available in Supplemental Appendix 1.)

### Data analysis

The estimated annual percentage change (EAPC) provides a summary measure of the trend in age-standardized rates (ASRs) over a specified period. A positive EAPC reflects an increasing trend, whereas a negative EAPC indicates a declining trend in the ASRs during the analyzed period (Supplemental Appendix 2).

#### Original XGBoost modeling strategy

Using the GBD 2021 data set for ID from 1990 to 2021, an XGBoost model was developed to predict incidence, prevalence, and DALY rates for ID. The model used variables such as sex, age, year, and the natural logarithm of the population size as input features, with the log-transformed incidence, prevalence, or DALY rates [log(Incidence,prevalence,orDALYrate+1)] of ID as the outputs. This transformation helped manage data skewness and ensured proper handling of zero values. The model used for the analysis took the following form:

$$\log\left({Outcome\;Rate}_{y,c,s,a}+1\right)\;∼\;s+a+y+{\left(\log\left(pnum\right)\right)}_{y,c,s,a}$$

where y stands for the calendar year; c indicates the nation or region; s represents sex (0 for female and 1 for male); a is the midpoint of the respective age group (e.g. 2 for <5 y, 7 for those aged 5–9 y, …, and 87 for aged 85+ y).

In this formulation, ${Outcome\;Rate}_{y,c,s,a}$ corresponded to the incidence, prevalence, or DALY rate for year y, nation or region c, sex s, and age group a. The term ${\left(\log\left(pnum\right)\right)}_{y,c,s,a}$ representing the natural logarithm of the population size corresponding to year y, nation or region c, sex s, and age group a, was included in the model as an adjustment factor. This ensured that variations in population size were accounted for, preventing population differences from disproportionately influencing the model outcomes. The natural logarithm transformation stabilized population effects and allowed the model to accurately capture trends independent of demographic shifts.

#### Iodized salt coverage-based XGBoost modeling strategy

To evaluate the impact of salt fortification programs, a revised XGBoost model was developed by replacing the year variable with iodized salt coverage. Data for iodized salt coverage were available for 66 countries at the national level for specific years between 1990 and 2021. However, these data had significant missing values across time and countries. To address this, a linear model was constructed to impute and forecast missing data: ${\left(iodized\;salt\;coverage\right)}_{y,c}\;∼\log\left(year\right)$. Using this model, iodized salt coverage was imputed for missing years from 1990 to 2021 and forested for the period 2022–2050 for these 66 countries. Given the high correlation between year and iodized salt coverage (*r* = 0.9), including both variables in the model would result in multicollinearity, which could distort the interpretation of the predictors. To avoid this issue, we excluded the year variable and retained only iodized salt coverage in the revised model. The revised XGBoost model used the following predictors: age, sex, log-transformed population size, and iodized salt coverage, although the outputs remained the same log-transformed outcome rates:

$$\log\left({Outcome\;Rate}_{y,c,s,a}+1\right)\;∼\;s+a+{\left(iodized\;salt\;coverage\right)}_{y,c}+{\left[\log\left(pnum\right)\right]}_{y,c,s,a}$$

The parameters retain the same definitions as in the original model, with the addition of the following: ${\left(iodized\;salt\;coverage\right)}_{y,c}$, percentage of households consuming adequately iodized salt (≥15 parts per million) for year y and country c.

#### Model training, validation, and performance evaluation

Both models (original and iodized salt coverage-based XGBoost models) followed identical training, validation, and evaluation processes. The XGBoost model used for this analysis builds decision trees iteratively to improve prediction accuracy. It uses an objective function comprising 2 components: a loss function that measures prediction errors and a regularization term that controls model complexity to prevent overfitting. The model grows trees sequentially, adding new trees to correct the prediction errors of the previous ones. The regularization term penalizes overly complex models by controlling the number of leaf nodes in each tree and the magnitude of their weights. This ensures that the model remains robust and avoids overfitting. (For more details on the XGBoost model used in this study, see Supplemental Appendix 3.)

To construct the XGBoost model, the data set was randomly divided into 70% for training and 30% for testing. Hyperparameter tuning was performed on the training set using 5-fold crossvalidation combined with a grid search approach. The evaluation metric used during tuning was root-mean-squared error (RMSE), which helped assess the performance of different hyperparameter configurations. After identifying the optimal set of hyperparameters, the final XGBoost model was trained and subsequently evaluated on the test set (Supplemental Appendix 4). Model performance was assessed by calculating both RMSE and Pearson correlation coefficient to compare the predicted values against the observed outcomes.

Following model validation, the trained XGBoost model was used to forecast ID incidence, prevalence, and DALY rates from 2022 to 2050. To quantify uncertainty in the predictions, 500 bootstrap samples were generated, and 95% uncertainty intervals (UI) were calculated based on the 2.5th and 97.5th percentiles of the forecasted outcomes.

To prevent overfitting during the training process, early stopping was implemented, which monitored the model’s performance on a validation set. In this case, out-of-bag data were used, and the early stopping criterion was set to halt training if no improvement was observed for 10 consecutive iterations. This helped not only reduce risk of overfitting but also optimize computational efficiency.

#### SHAP analysis

SHAP values were used to interpret the contributions of predictors in both models, quantifying the importance of age, sex, population size, and iodized salt coverage (in the iodized salt coverage-based XGBoost model) in influencing ID burden. By calculating mean absolute SHAP values, the analysis assessed the overall contribution of each predictor, although SHAP values also clarified how these factors influenced model predictions. This approach enhanced the interpretability of the model and provided valuable insights into the key drivers of ID incidence, prevalence, and DALYs (more details about SHAP can be found in Supplemental Appendix 5).

#### APC analysis

An APC model was applied to both the observed data (1990–2021) and the forecasted results of ID from the original XGBoost model for the period 2022–2050. This allowed the decomposition of the effects of age, period, and cohort on the incidence, prevalence, and DALY rates of ID. Rate ratios (RRs) were calculated to compare these rates across different periods and birth cohorts, relative to a reference group (Supplemental Appendix 6).

At the national level, the weighted correlations between SDI and the ASPR, ASIR, and ASDR of ID were analyzed, using prevalent cases, incident cases, and DALYs as weights to explore how socioeconomic factors influence the national burden of ID. Similarly, for the subset of 66 countries with available iodized salt coverage data, we analyzed the weighted correlations between iodized salt coverage and the ASPR, ASIR, and ASDR of ID, using the same weighting approach.

The APC analysis was carried out using the APC Web Tool [21], with data preprocessing and visualization completed in R (version 4.4.2). The XGBoost model and SHAP analysis were implemented using the xgboost and SHAPforxgboost packages, respectively [22,23].

## Results

### Model tuning and validation, and sensitivity analysis

To determine the optimal hyperparameters and evaluate the predictive performance of the XGBoost model, the data were split into 70% for training and 30% for testing. A grid search was performed on the training set to explore combinations of 3 key hyperparameters: *nrounds* (number of boosting rounds), *eta* (learning rate), and *max_depth* (maximum tree depth). The *max_depth* controls the depth of each tree; deeper trees can capture more complex patterns but may increase risk of overfitting. The learning rate (*eta*) determines how quickly the model adapts to new patterns, influencing the convergence speed and overall stability. The *nrounds* parameter defines how many boosting iterations the model will go through, impacting both its learning potential and the likelihood of overfitting.

A 5-fold crossvalidation was applied, using RMSE as the performance metric to identify the best hyperparameter combination. The configuration with the lowest RMSE was selected as optimal, and this setup was then used to predict ID incidence, prevalence, and DALY rates for the years 2022 to 2050. For the original XGBoost model, the RMSE values on the test set, were 5.95/100,000 for incidence, 73.99/100,000 for prevalence, and 0.95/100,000 for DALYs, with Pearson correlation coefficients between observed and predicted values being 1.00 (*P* < 0.001) across all metrics, indicating excellent predictive accuracy (Supplemental Figure 1). Similarly, for the iodized salt coverage-based XGBoost model, the RMSE values on the test set were 8.34/100,000 for incidence, 113.24/100,000 for prevalence, and 1.33/100,000 for DALYs. Pearson correlation coefficients for this model were also 1.00 (*P* < 0.001) across all metrics (Supplemental Figure 1). Despite being comparable, the iodized salt coverage-based model exhibited slightly higher RMSE values for all metrics, indicating marginally lower predictive performance than the original model. Given the slightly better overall accuracy and reliability of the original XGBoost model, its predictions were used as the primary source for the subsequent analyses and results presented in this study. Nonetheless, the iodized salt coverage-based model provided valuable complementary insights into the potential impact of salt fortification programs.

### Overall burden of ID

#### Incidence of ID

**FIGURE 1:**
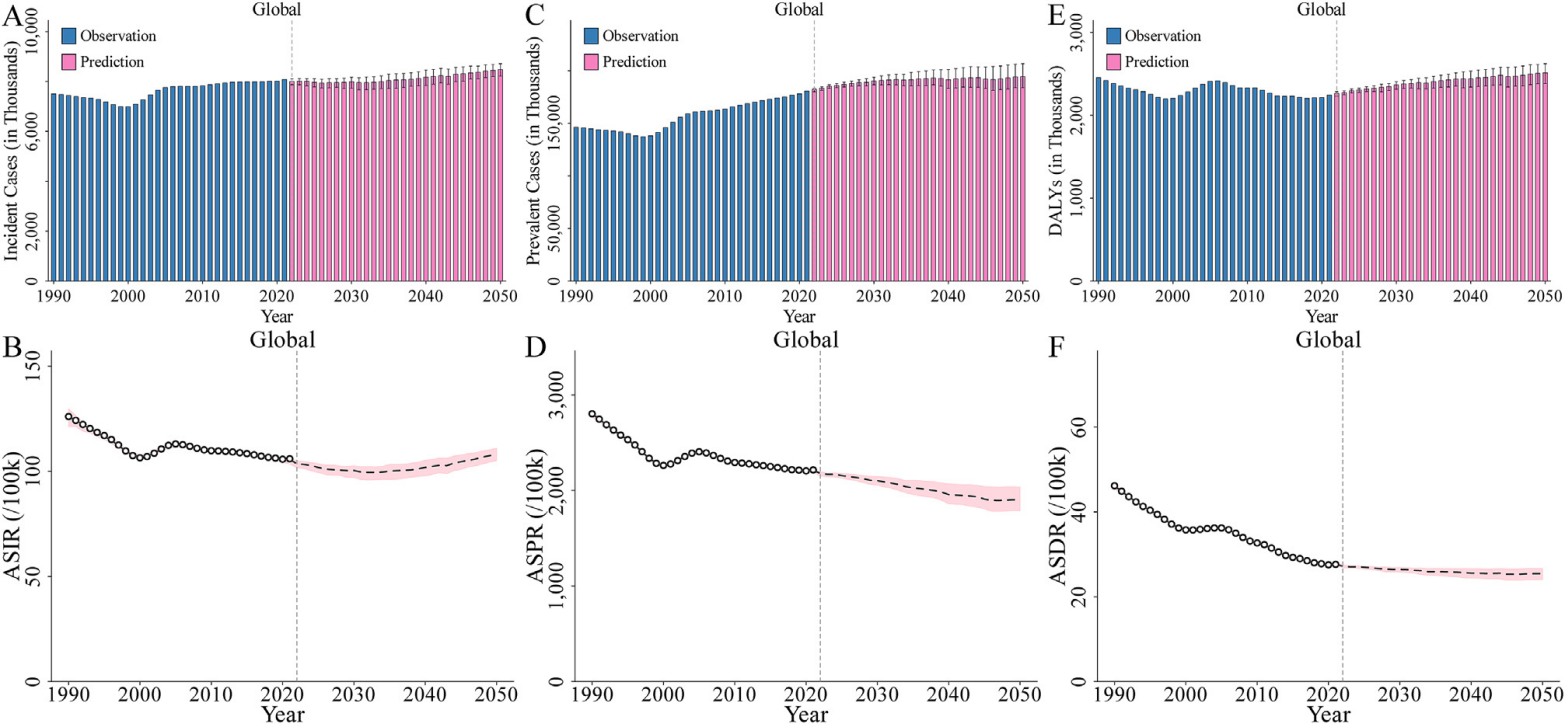
Number and ASR of ID from 1990 to 2050 at the global level. (A) Incident cases; (B) ASIR; (C) prevalent cases; (D) ASPR; (E) DALYs; (F) ASDR. ASDR, age-standardized DALY rate; ASIR, age-standardized incidence rate; ASPR, age-standardized prevalence rate; ASR, age-standardized rate; DALY, disability-adjusted life year; ID, iodine deficiency.

**FIGURE 2:**
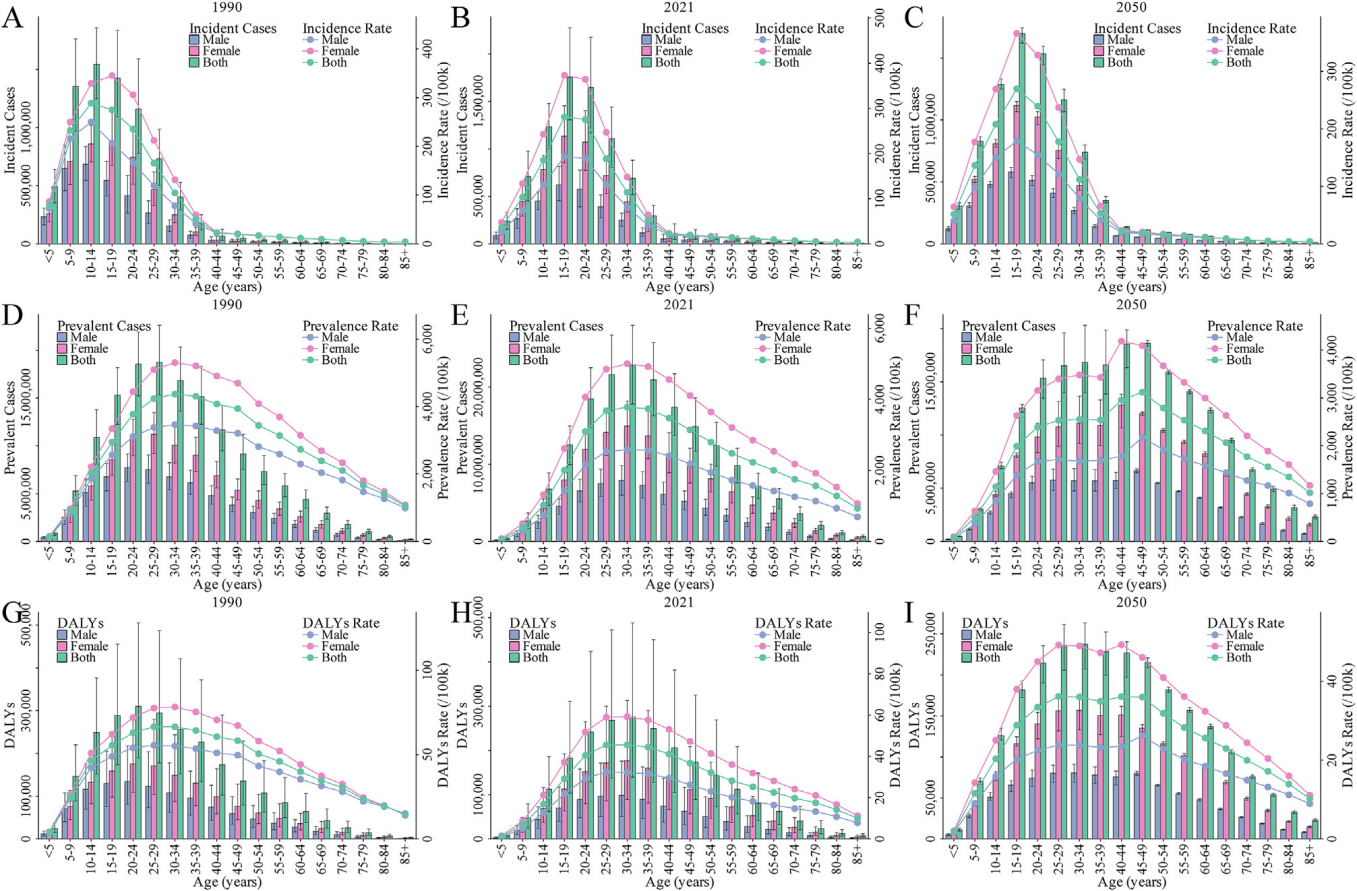
Number and rate of ID in 1990, 2021, and 2050 at the global level by gender and age groups. (A–C) Incident case and incidence rate in 1990 (A), 2021 (B), and 2050 (C). (D–F) Prevalent case and prevalence rate in 1990 (D), 2021 (E), and 2050 (F). (G–I) DALYs and DALY rate in 1990 (G), 2021 (H), and 2050 (I). DALY, disability-adjusted life year; ID, iodine deficiency.

In 1990, the global incident cases of ID were 7.51 million, rising to 8.08 million in 2021 (a 7.65% increase from 1990) and projected to reach 8.48 million by 2050 (a 4.92% increase from 2021). The ASIR decreased from 126.11/100,000 in 1990 to 105.99/100,000 in 2021 (a 15.96% decrease from 1990), with a slight increase projected to 108.20/100,000 by 2050 (a 2.09% increase from 2021). The EAPC of ASIR from 1990 to 2021 was −0.40 (95% CI: −0.51, −0.28), whereas from 2021 to 2050, it is expected to be 0.14 (95% CI: 0.04, 0.23) (Table 1, Figure 1A, B, and Supplemental Table 1). Higher incidence rates are particularly observed in individuals younger than 40 y, especially within the 10–30-y age range. Across nearly all age groups, females had higher incidence rates than males (Figure 2, Supplemental Figures 2A–C and **3**B, and Supplemental Table 2). For 1990, 2021, and 2050, the ASIR for males compared with females was 103.24/100,000 compared with 149.66/100,000, 75.53/100,000 compared with 137.79/100,000, and 76.26/100,000 compared with 142.08/100,000, respectively (Table 1 and Supplemental Table 3).

**TABLE 1:** Number, ASR, EAPC, and change percentage for incidence, prevalence, and DALYs of ID.

Measure	Location	Number in thousands (ASR, per 100,000)			EAPC (%)		Change of all ages number (ASR) (%)	
		1990	2021	2050	1990–2021	2021–2050	1990–2021	2021–2050
Incidence	Global	7505.82 (126.11)	8079.84 (105.99)	8477.40 (108.20)	−0.40 (−0.51 to −0.28)	0.14 (0.04 to 0.23)	7.65 (−15.96)	4.92 (2.09)
	Sex							
	Male	3120.22 (103.24)	2940.96 (75.53)	3084.79 (76.26)	−0.74 (−0.91 to −0.57)	0.12 (0.03 to 0.20)	−5.75 (−26.84)	4.89 (0.97)
	Female	4385.59 (149.66)	5138.88 (137.79)	5392.62 (142.08)	−0.18 (−0.26 to −0.09)	0.16 (0.06 to 0.25)	17.18 (−7.93)	4.94 (3.11)
	Region							
	Andean Latin America	2.16 (5.33)	3.04 (4.54)	3.84 (4.49)	−0.59 (−0.66 to −0.53)	−0.03 (−0.03 to −0.02)	40.74 (−14.69)	26.41 (−1.17)
	Australasia	2.55 (12.88)	3.40 (12.11)	3.97 (12.07)	−0.21 (−0.22 to −0.20)	−0.02 (−0.02 to −0.01)	33.28 (−5.98)	16.88 (−0.33)
	Caribbean	13.22 (33.79)	12.50 (27.33)	10.92 (27.21)	−1.03 (−1.12 to −0.93)	−0.01 (−0.01 to 0.00)	−5.49 (−19.11)	−12.62 (−0.45)
	Central Asia	21.88 (27.54)	17.56 (18.43)	19.76 (18.31)	−2.05 (−2.40 to −1.69)	−0.02 (−0.03 to 0.00)	−19.75 (−33.07)	12.54 (−0.67)
	Central Europe	18.46 (15.57)	10.73 (11.50)	7.84 (11.43)	−1.16 (−1.23 to −1.09)	−0.02 (−0.02 to −0.02)	−41.87 (−26.16)	−26.96 (−0.62)
	Central Latin America	57.04 (29.36)	71.40 (28.75)	77.11 (28.55)	−0.15 (−0.24 to −0.05)	−0.02 (−0.02 to −0.02)	25.18 (−2.06)	7.99 (−0.70)
	Central Sub-Saharan Africa	417.60 (578.68)	727.03 (405.20)	978.08 (365.17)	−0.94 (−1.27 to −0.61)	−0.38 (−0.43 to −0.32)	74.10 (−29.98)	34.53 (−9.88)
	East Asia	915.37 (65.08)	791.51 (67.59)	569.23 (67.57)	0.26 (−0.14 to 0.65)	−0.02 (−0.03 to −0.01)	−13.53 (3.85)	−28.08 (−0.03)
	Eastern Europe	23.30 (11.41)	19.05 (11.55)	15.28 (11.55)	−0.39 (−0.74 to −0.04)	−0.01 (−0.01 to 0.00)	−18.26 (1.24)	−19.75 (0.01)
	Eastern Sub-Saharan Africa	533.74 (233.96)	965.56 (185.94)	1249.59 (140.76)	−0.73 (−1.06 to −0.39)	−0.83 (−0.95 to −0.70)	80.90 (−20.52)	29.42 (−24.30)
	High-income Asia Pacific	29.04 (17.24)	21.71 (14.51)	16.82 (14.42)	−0.58 (−0.61 to −0.55)	−0.02 (−0.02 to −0.02)	−25.24 (−15.84)	−22.49 (−0.64)
	High-income North America	36.03 (13.36)	43.84 (13.19)	45.85 (13.20)	−0.03 (−0.05 to −0.01)	0.00 (0.00 to 0.00)	21.67 (−1.30)	4.59 (0.06)
	North Africa and Middle East	254.33 (61.64)	274.07 (42.30)	317.10 (40.56)	−1.76 (−1.97 to −1.56)	−0.07 (−0.10 to −0.04)	7.76 (−31.37)	15.70 (−4.12)
	Oceania	0.51 (7.33)	0.62 (4.35)	0.96 (4.10)	−1.98 (−2.24 to −1.71)	−0.19 (−0.22 to −0.16)	21.75 (−40.61)	53.41 (−5.66)
	South Asia	4295.48 (323.11)	4246.75 (207.06)	3580.18 (209.45)	−1.24 (−1.60 to −0.88)	0.06 (0.03 to 0.09)	−1.13 (−35.92)	−15.70 (1.15)
	Southeast Asia	393.99 (72.99)	289.47 (40.63)	287.34 (42.52)	−2.03 (−2.23 to −1.83)	0.17 (0.16 to 0.19)	−26.53 (−44.34)	−0.74 (4.67)
	Southern Latin America	5.15 (10.14)	5.10 (8.04)	5.30 (8.08)	−0.83 (−0.89 to −0.78)	0.03 (0.02 to 0.03)	−1.03 (−20.66)	3.96 (0.43)
	Southern Sub-Saharan Africa	49.70 (79.27)	46.84 (54.73)	55.85 (52.60)	−0.85 (−1.08 to −0.63)	−0.11 (−0.13 to −0.09)	−5.76 (−30.96)	19.25 (−3.90)
	Tropical Latin America	11.49 (6.96)	13.77 (6.27)	13.08 (6.26)	−0.44 (−0.47 to −0.41)	−0.01 (−0.01 to −0.01)	19.84 (−9.91)	−5.02 (−0.20)
	Western Europe	220.51 (61.87)	168.77 (49.14)	157.25 (48.84)	−0.90 (−1.03 to −0.77)	−0.03 (−0.03 to −0.02)	−23.47 (−20.57)	−6.83 (−0.61)
	Western Sub-Saharan Africa	204.27 (92.10)	347.14 (62.20)	610.44 (56.83)	−1.75 (−1.91 to −1.58)	−0.28 (−0.32 to −0.24)	69.94 (−32.46)	75.85 (−8.64)
Prevalence	Global	146,418.05 (2801.80)	180,812.70 (2213.98)	194,507.42 (1900.01)	−0.59 (−0.73 to −0.45)	−0.56 (−0.59 to −0.54)	23.49 (−20.98)	7.57 (−14.18)
	Sex							
	Male	61,215.65 (2323.28)	63,105.58 (1542.43)	66,686.17 (1298.35)	−1.03 (−1.23 to −0.83)	−0.59 (−0.63 to −0.55)	3.09 (−33.61)	5.67 (−15.82)
	Female	85,202.40 (3287.18)	117,707.12 (2891.38)	127,821.25 (2519.03)	−0.33 (−0.43 to −0.23)	−0.53 (−0.56 to −0.50)	38.15 (−12.04)	8.59 (−12.88)
	Region							
	Andean Latin America	30.73 (90.53)	50.05 (74.59)	68.94 (72.10)	−0.74 (−0.82 to −0.66)	−0.13 (−0.14 to −0.13)	62.89 (−17.61)	37.74 (−3.33)
	Australasia	47.11 (219.63)	69.36 (205.79)	84.31 (205.60)	−0.23 (−0.24 to −0.21)	−0.01 (−0.01 to −0.01)	47.22 (−6.30)	21.55 (−0.09)
	Caribbean	240.92 (701.71)	264.87 (538.05)	278.14 (545.79)	−1.29 (−1.42 to −1.17)	0.03 (0.03 to 0.04)	9.94 (−23.32)	5.01 (1.44)
	Central Asia	415.59 (615.23)	370.29 (384.15)	464.62 (378.50)	−2.41 (−2.81 to −2.00)	−0.07 (−0.07 to −0.06)	−10.90 (−37.56)	25.48 (−1.47)
	Central Europe	386.22 (296.49)	258.49 (209.70)	201.21 (196.18)	−1.32 (−1.41 to −1.23)	−0.23 (−0.26 to −0.21)	−33.07 (−29.27)	−22.16 (−6.45)
	Central Latin America	934.53 (607.13)	1604.11 (609.20)	2080.71 (598.69)	−0.09 (−0.20 to 0.02)	−0.04 (−0.05 to −0.03)	71.65 (0.34)	29.71 (−1.73)
	Central Sub-Saharan Africa	7841.20 (17,290.43)	14,164.39 (11,855.67)	26,065.19 (10,607.98)	−0.92 (−1.30 to −0.55)	−0.41 (−0.42 to −0.40)	80.64 (−31.43)	84.02 (−10.52)
	East Asia	17,618.79 (1412.92)	27,548.68 (1592.36)	25,369.74 (1511.85)	0.57 (0.15 to 1.00)	−0.24 (−0.25 to −0.22)	56.36 (12.70)	−7.91 (−5.06)
	Eastern Europe	542.44 (232.07)	516.54 (239.18)	443.42 (237.39)	−0.39 (−0.77 to −0.01)	−0.03 (−0.04 to −0.03)	−4.77 (3.07)	−14.16 (−0.75)
	Eastern Sub-Saharan Africa	9068.62 (6375.92)	16,101.72 (4614.38)	29,030.56 (3655.96)	−1.04 (−1.40 to −0.67)	−0.80 (−0.82 to −0.78)	77.55 (−27.63)	80.29 (−20.77)
	High-income Asia Pacific	569.05 (302.03)	524.40 (250.41)	429.57 (239.60)	−0.64 (−0.67 to −0.61)	−0.16 (−0.16 to −0.16)	−7.85 (−17.09)	−18.08 (−4.31)
	High-income North America	688.25 (230.01)	917.42 (226.54)	1012.29 (226.87)	−0.04 (−0.06 to −0.02)	0.00 (0.00 to 0.00)	33.30 (−1.51)	10.34 (0.14)
	North Africa and Middle East	4545.13 (1474.46)	5364.07 (854.53)	7518.50 (801.52)	−2.45 (−2.71 to −2.20)	−0.18 (−0.20 to −0.16)	18.02 (−42.04)	40.16 (−6.20)
	Oceania	7.24 (129.17)	9.47 (75.31)	14.29 (66.02)	−2.04 (−2.31 to −1.77)	−0.46 (−0.47 to −0.45)	30.89 (−41.70)	50.90 (−12.33)
	South Asia	86,509.56 (8746.81)	95,152.80 (5021.98)	118,800.97 (4872.25)	−1.55 (−1.92 to −1.18)	−0.10 (−0.14 to −0.06)	9.99 (−42.59)	24.85 (−2.98)
	Southeast Asia	6611.98 (1572.68)	5782.17 (774.12)	6933.41 (799.23)	−2.46 (−2.64 to −2.28)	0.10 (0.09 to 0.11)	−12.55 (−50.78)	19.91 (3.24)
	Southern Latin America	86.07 (174.18)	96.59 (135.66)	110.26 (135.69)	−0.89 (−0.95 to −0.84)	0.00 (0.00 to 0.00)	12.22 (−22.11)	14.16 (0.02)
	Southern Sub-Saharan Africa	838.09 (1796.10)	852.80 (1058.40)	1173.88 (984.19)	−1.29 (−1.57 to −1.02)	−0.25 (−0.25 to −0.24)	1.76 (−41.07)	37.65 (−7.01)
	Tropical Latin America	169.58 (117.21)	257.77 (106.04)	287.28 (105.70)	−0.42 (−0.45 to −0.39)	−0.01 (−0.01 to −0.01)	52.00 (−9.53)	11.45 (−0.31)
	Western Europe	5956.96 (1384.83)	5474.77 (1087.24)	5250.35 (1018.86)	−0.96 (−1.10 to −0.81)	−0.22 (−0.23 to −0.21)	−8.09 (−21.49)	−4.10 (−6.29)
	Western Sub-Saharan Africa	3309.97 (2148.65)	5431.93 (1376.82)	10,425.05 (1122.16)	−1.98 (−2.16 to −1.80)	−0.69 (−0.71 to −0.68)	64.11 (−35.92)	91.92 (−18.50)
DALYs	Global	2455.26 (46.19)	2246.33 (27.67)	2513.98 (25.51)	−1.56 (−1.66 to −1.45)	−0.27 (−0.30 to −0.24)	−8.51 (−40.10)	11.91 (−7.80)
	Sex							
	Male	1072.08 (39.98)	813.99 (19.99)	888.01 (17.87)	−2.04 (−2.19 to −1.89)	−0.36 (−0.39 to −0.32)	−24.07 (−50.01)	9.09 (−10.59)
	Female	1383.18 (52.52)	1432.34 (35.43)	1625.97 (33.42)	−1.25 (−1.33 to −1.17)	−0.21 (−0.24 to −0.18)	3.55 (−32.54)	13.52 (−5.68)
	Region							
	Andean Latin America	0.69 (2.05)	0.65 (0.97)	0.90 (0.94)	−3.20 (−3.71 to −2.70)	−0.11 (−0.11 to −0.10)	−5.59 (−52.68)	38.12 (−2.82)
	Australasia	0.50 (2.34)	0.74 (2.20)	0.89 (2.20)	−0.22 (−0.23 to −0.21)	−0.01 (−0.01 to 0.00)	46.99 (−5.95)	21.06 (−0.03)
	Caribbean	4.11 (11.86)	4.64 (9.47)	4.85 (9.58)	−1.12 (−1.23 to −1.00)	0.03 (0.03 to 0.04)	13.02 (−20.12)	4.55 (1.13)
	Central Asia	6.67 (9.82)	5.72 (5.94)	7.12 (5.85)	−2.52 (−2.98 to −2.06)	−0.07 (−0.07 to −0.06)	−14.27 (−39.52)	24.48 (−1.47)
	Central Europe	4.83 (3.72)	2.91 (2.38)	2.23 (2.16)	−1.92 (−2.22 to −1.63)	−0.38 (−0.41 to −0.35)	−39.75 (−35.97)	−23.36 (−9.51)
	Central Latin America	13.65 (8.80)	21.70 (8.25)	27.91 (8.09)	−0.66 (−1.10 to −0.21)	0.02 (−0.02 to 0.05)	58.92 (−6.21)	28.65 (−1.94)
	Central Sub-Saharan Africa	88.19 (190.84)	165.36 (135.41)	314.77 (126.67)	−0.83 (−1.21 to −0.46)	−0.24 (−0.25 to −0.24)	87.51 (−29.05)	90.35 (−6.45)
	East Asia	233.07 (18.59)	294.66 (17.10)	266.83 (16.25)	0.10 (−0.26 to 0.45)	−0.24 (−0.25 to −0.22)	26.42 (−8.01)	−9.45 (−5.00)
	Eastern Europe	10.40 (4.42)	9.90 (4.56)	8.40 (4.47)	−0.43 (−0.84 to −0.02)	−0.07 (−0.08 to −0.07)	−4.81 (3.12)	−15.17 (−1.91)
	Eastern Sub-Saharan Africa	116.39 (78.32)	197.49 (54.92)	364.09 (45.29)	−1.17 (−1.51 to −0.83)	−0.65 (−0.67 to −0.63)	69.68 (−29.87)	84.37 (−17.54)
	High-income Asia Pacific	6.10 (3.24)	5.58 (2.69)	4.62 (2.57)	−0.63 (−0.67 to −0.60)	−0.18 (−0.18 to −0.17)	−8.63 (−16.99)	−17.25 (−4.39)
	High-income North America	7.34 (2.46)	9.67 (2.41)	10.64 (2.41)	−0.05 (−0.07 to −0.03)	0.00 (0.00 to 0.00)	31.85 (−1.99)	10.05 (0.19)
	North Africa and Middle East	92.03 (29.40)	101.09 (16.07)	140.08 (15.08)	−2.48 (−2.68 to −2.28)	−0.18 (−0.20 to −0.16)	9.85 (−45.33)	38.56 (−6.19)
	Oceania	0.16 (2.89)	0.20 (1.61)	0.33 (1.50)	−2.08 (−2.36 to −1.80)	−0.27 (−0.28 to −0.26)	26.66 (−44.15)	61.62 (−7.03)
	South Asia	1632.38 (158.60)	1198.79 (62.87)	1497.63 (64.56)	−2.88 (−3.03 to −2.73)	0.15 (0.10 to 0.20)	−26.56 (−60.36)	24.93 (2.68)
	Southeast Asia	118.98 (27.77)	83.98 (11.29)	98.82 (11.47)	−3.41 (−3.98 to −2.83)	0.05 (0.04 to 0.06)	−29.42 (−59.37)	17.67 (1.62)
	Southern Latin America	0.92 (1.87)	1.03 (1.45)	1.17 (1.45)	−0.89 (−0.95 to −0.83)	0.00 (0.00 to 0.00)	11.71 (−22.18)	13.51 (−0.01)
	Southern Sub-Saharan Africa	9.88 (20.89)	9.59 (11.86)	13.60 (11.47)	−1.55 (−1.76 to −1.34)	−0.10 (−0.10 to −0.09)	−2.97 (−43.24)	41.81 (−3.24)
	Tropical Latin America	2.01 (1.39)	2.73 (1.12)	3.01 (1.12)	−0.67 (−0.71 to −0.62)	−0.01 (−0.01 to −0.01)	35.68 (−18.84)	10.28 (−0.43)
	Western Europe	63.29 (14.77)	57.93 (11.60)	54.78 (10.88)	−0.95 (−1.10 to −0.81)	−0.21 (−0.22 to −0.21)	−8.47 (−21.45)	−5.43 (−6.19)
	Western Sub-Saharan Africa	43.66 (27.36)	71.97 (17.63)	147.49 (15.63)	−2.03 (−2.21 to −1.85)	−0.41 (−0.42 to −0.40)	64.84 (−35.58)	104.92 (−11.33)

Abbreviations: ASR, age-standardized rate; DALY, disability-adjusted life year; EAPC, estimated annual percentage change; ID, iodine deficiency.

**FIGURE 3:**
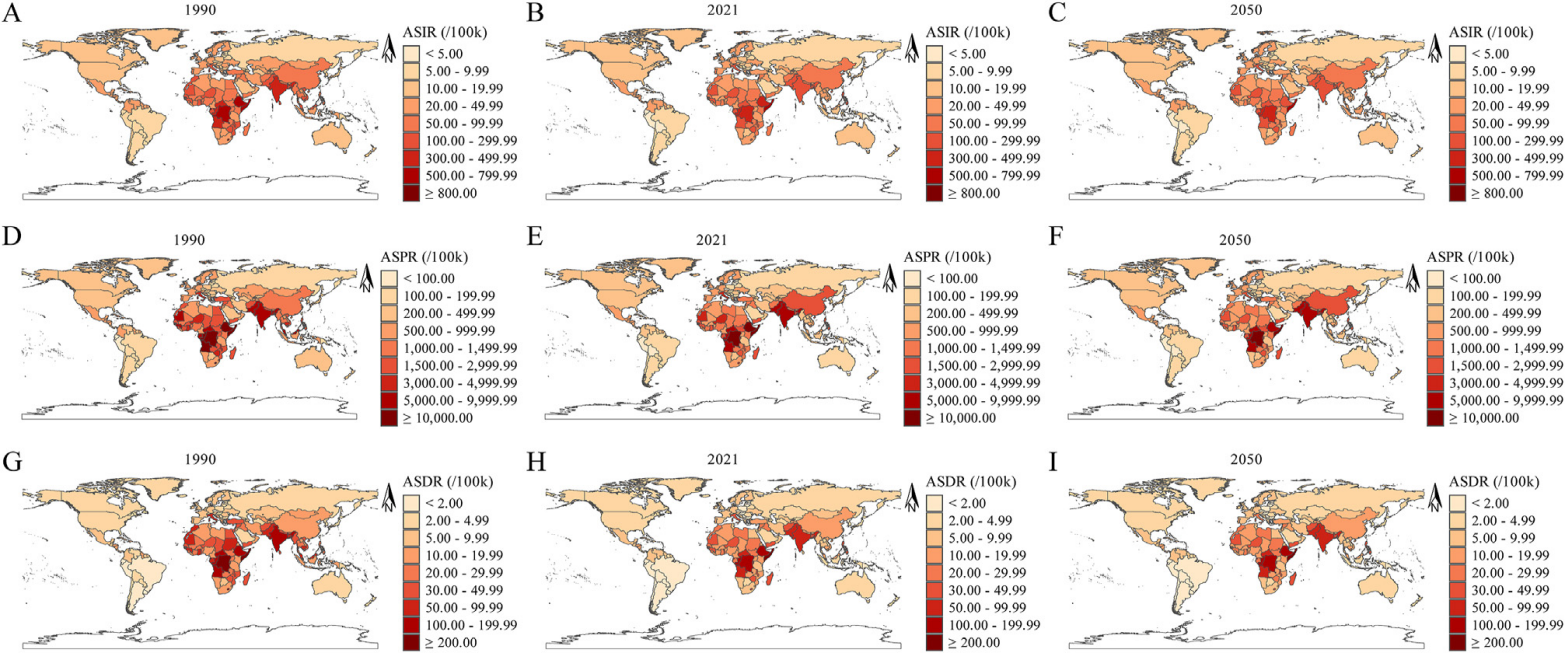
ASR of ID at the national level in 1990, 2021, and 2050. (A–C) ASIR in 1990 (A), 2021 (B), and 2050 (C). (D–F) ASPR in 1990 (D), 2021 (E), and 2050 (F). (G–I) ASDR in 1990 (G), 2021 (H), and 2050 (I). ASDR, age-standardized DALY rate; ASIR, age-standardized incidence rate; ASPR, age-standardized prevalence rate; ASR, age-standardized rate; DALY, disability-adjusted life year; ID, iodine deficiency.

**FIGURE 4:**
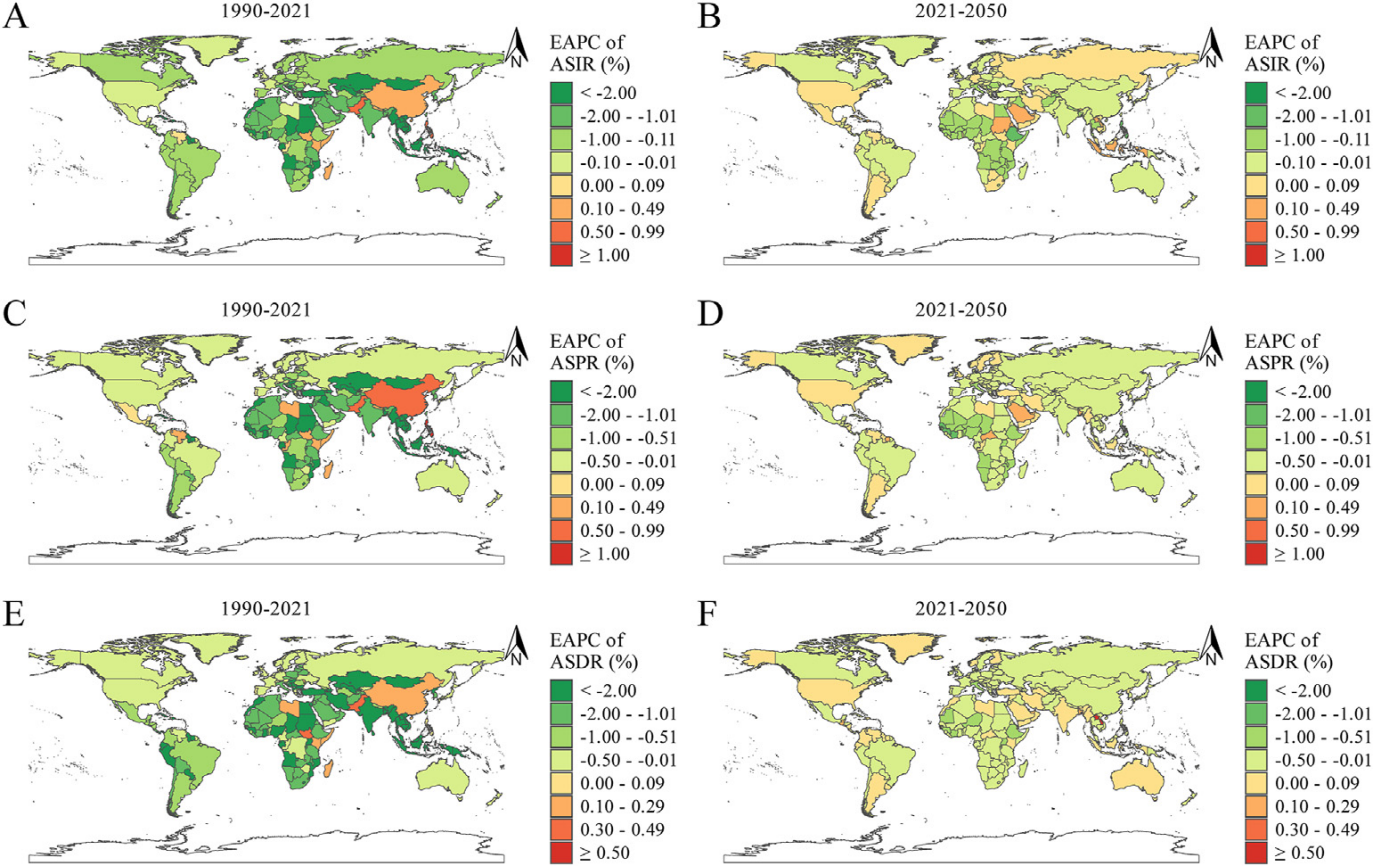
EAPC in ASR of ID at the national level from 1990 to 2021 and from 2021 to 2050. (A, B) EAPC in ASIR from 1990 to 2021 (A) and from 2021 to 2050 (B). (C, D) EAPC in ASPR from 1990 to 2021 (C) and from 2021 to 2050 (D). (E, F) EAPC in ASDR from 1990 to 2021 (C) and from 2021 to 2050 (D). ASDR, age-standardized DALY rate; ASIR, age-standardized incidence rate; ASPR, age-standardized prevalence rate; ASR, age-standardized rate; DALY, disability-adjusted life year; EAPC, estimated annual percentage change; ID, iodine deficiency.

At the regional level, Central Sub-Saharan Africa had the highest ASIR in 1990 (578.68/100,000), followed by South Asia (323.11/100,000) and Eastern Sub-Saharan Africa (233.96/100,000). In 2021, Central Sub-Saharan Africa remained the highest (405.20/100,000), with South Asia and Eastern Sub-Saharan Africa at 207.06/100,000 and 185.94/100,000, respectively. Projections for 2050 suggest Central Sub-Saharan Africa will continue to lead (365.17/100,000), followed by South Asia (209.45/100,000) and Eastern Sub-Saharan Africa (140.76/100,000) (Table 1, Supplemental Table 3, and Figure 3A–C). From 1990 to 2021, the highest EAPC of ASIR was observed in East Asia (0.26%), whereas from 2021 to 2050, the highest was projected to be in Southeast Asia (0.17%) (Table 1, Supplemental Table 4, and Figure 4A, B).

At the national level, the highest ASIRs in 1990 were in Equatorial Guinea (873.69/100,000), the Democratic Republic of the Congo (665.94/100,000), and Somalia (651.47/100,000). In 2021, Somalia (725.59/100,000), the Democratic Republic of the Congo (487.60/100,000), and Djibouti (474.30/100,000) topped the list. By 2050, the highest ASIRs are projected in Somalia (699.42/100,000), Djibouti (488.73/100,000), and the Democratic Republic of the Congo (448.53/100,000) (Table 2, Supplemental Table 3, Figure 3A–C, and Supplemental Movie 1). From 1990 to 2021, the largest EAPCs of ASIR were in the Philippines (1.00%), Pakistan (0.65%), and Nepal (0.32%), whereas between 2021 and 2050, they are expected in Comoros (0.51%), Indonesia (0.42%), and Bhutan (0.37%) (Figure 4A, B and Supplemental Table 4).

**TABLE 2:** Top 3 nations with largest ASRs of ID in 1990, 2021, and 2050.

Year	ASIR (per 100,000)	ASPR (per 100,000)	ASDR (per 100,000)
1990	Equatorial Guinea (873.69)	Equatorial Guinea (27,675.35)	Equatorial Guinea (301.21)
	Democratic Republic of the Congo (665.94)	Democratic Republic of the Congo (20,341.94)	Democratic Republic of the Congo (223.35)
	Somalia (651.47)	Somalia (18,710.07)	Somalia (222.27)
2021	Somalia (725.59)	Somalia (20,808.11)	Somalia (241.52)
	Democratic Republic of the Congo (487.60)	Democratic Republic of the Congo (14,426.07)	Democratic Republic of the Congo (165.16)
	Djibouti (474.30)	Djibouti (13,035.45)	Djibouti (144.96)
2050	Somalia (699.42)	Somalia (20,970.08)	Somalia (241.54)
	Djibouti (488.73)	Democratic Republic of the Congo (13,563.43)	Democratic Republic of the Congo (159.54)
	Democratic Republic of the Congo (448.53)	Djibouti (13,133.61)	Djibouti (145.84)

Abbreviations: ASDR, age-standardized DALY rate; ASIR, age-standardized incidence rate; ASPR, age-standardized prevalence rate; ASR, age-standardized rate; DALYs, disability-adjusted life years; ID, iodine deficiency.

#### Prevalence of ID

In 1990, the global prevalent cases of ID were 146.42 million, increasing to 180.81 million by 2021 (a 23.49% increase from 1990) and projected to reach 194.51 million by 2050 (a 7.57% increase from 2021). The ASPR dropped from 2801.80/100,000 in 1990 to 2213.98/100,000 in 2021 (a 20.98% decrease from 1990) and is expected to decrease further to 1900.01/100,000 by 2050 (a 14.18% decrease from 2021). The EAPC of the ASPR from 1990 to 2021 was −0.59% (95% CI: −0.73%, −0.45%) and from 2021 to 2050 to be −0.56% (95% CI: −0.59%, −0.54%) (Table 1, Figure 1C, D, and Supplemental Table 1). The prevalence was higher among individuals aged 5 y and older, with females exhibiting higher rates than males across nearly all age groups. In 1990, 2021, and 2050, the ASPR for males compared with females were 2323.28/100,000 compared with 3287.18/100,000, 1542.43/100,000 compared with 2891.38/100,000, and 1298.35/100,000 compared with 2519.03/100,000, respectively (Table 1, Figure 2D–F, Supplemental Figure 2D–F, Supplemental Tables 2 and 4).

At the regional level, in 1990, Central Sub-Saharan Africa had the highest ASPR (17,290.43/100,000), followed by South Asia (8746.81/100,000) and Eastern Sub-Saharan Africa (6375.92/100,000). By 2021, these regions still had the highest ASPRs, with Central Sub-Saharan Africa at 11,855.67/100,000, South Asia at 5021.98/100,000, and Eastern Sub-Saharan Africa at 4614.38/100,000. By 2050, Central Sub-Saharan Africa is projected to maintain the highest ASPR (10,607.98/100,000), followed by South Asia (4872.25/100,000) and Eastern Sub-Saharan Africa (3655.96/100,000) (Table 1, Supplemental Table 3, and Figure 3D–F). The largest EAPC of ASPR from 1990 to 2021 was observed in East Asia (0.57%, 95% CI: 0.15%, 1.00%), whereas Southeast Asia is expected to have the highest EAPC from 2021 to 2050 (0.10%, 95% CI: 0.09%, 0.11%) (Table 1, Supplemental Table 4, and Figure 4C, D).

At the national level, in 1990, the countries with the highest ASPR were Equatorial Guinea (27,675.35/100,000), the Democratic Republic of the Congo (20,341.94/100,000), and Somalia (18,710.07/100,000). By 2021, the top 3 countries were Somalia (20,808.11/100,000), the Democratic Republic of the Congo (14,426.07/100,000), and Djibouti (13,035.45/100,000). Projections for 2050 indicate that Somalia (20,970.08/100,000), the Democratic Republic of the Congo (13,563.43/100,000), and Djibouti (13,133.61/100,000) will continue to have the highest ASPRs (Table 2, Supplemental Table 3, Figure 3D–F, and Supplemental Movie 2). From 1990 to 2021, the country with the largest EAPC of ASPR was the Philippines (1.02%), whereas from 2021 to 2050, the United Arab Emirates is expected to have the highest EAPC (0.25%) (Figure 4C, D and Supplemental Table 4).

#### DALYs of ID

In 1990, the global DALYs due to ID were 2.46 million, decreasing to 2.25 million by 2021 (an 8.51% decrease from 1990), and projected to rise to 2.51 million by 2050 (an 11.91% increase from 2021). The ASDR dropped from 46.19/100,000 in 1990 to 27.67/100,000 in 2021 (a 40.10% decrease from 1990) and is expected to decline further to 25.51/100,000 by 2050 (a 7.80% decrease from 2021). The corresponding EAPC of ASDR between 1990 and 2021 was −1.56% (95% CI: −1.66%, −1.45%), and from 2021 to 2050, the EAPC is projected to be −0.27% (95% CI: −0.30%, −0.24%). Similar to the prevalence distribution, higher DALY rates were observed in individuals aged 5 y and older, with females showing higher rates than males across nearly all age groups (Table 1, Figure 2G–I, Supplemental Figure 2G–I, Supplemental Tables 2 and 4).

At the regional level, in 1990, the highest ASDRs were recorded in Central Sub-Saharan Africa (190.84/100,000), South Asia (158.60/100,000), and Eastern Sub-Saharan Africa (78.32/100,000). By 2021, Central Sub-Saharan Africa (135.41/100,000), South Asia (62.87/100,000), and Eastern Sub-Saharan Africa (54.92/100,000) remained the regions with the highest ASDRs. Projections for 2050 indicate that these regions will still lead, with Central Sub-Saharan Africa at 126.67/100,000, South Asia at 64.56/100,000, and Eastern Sub-Saharan Africa at 45.29/100,000 (Table 1, Supplemental Table 3, and Figure 3G–I). The largest EAPC of ASDR between 1990 and 2021 was observed in East Asia (0.10%), whereas the region with the highest projected EAPC between 2021 and 2050 is South Asia (0.15%) (Table 1, Supplemental Table 4, and Figure 4E, F).

At the national level, in 1990, the highest ASDRs were found in Equatorial Guinea (301.21/100,000), the Democratic Republic of the Congo (223.35/100,000), and Somalia (222.27/100,000). By 2021, Somalia (241.52/100,000), the Democratic Republic of the Congo (165.16/100,000), and Djibouti (144.96/100,000) had the highest ASDRs. Projections for 2050 suggested that Somalia (241.54/100,000), the Democratic Republic of the Congo (159.54/100,000), and Djibouti (145.84/100,000) will continue to have the highest rates (Table 2, Supplemental Table 3, Figure 3G–I, and Supplemental Movie 3). The country with the largest EAPC of ASDR between 1990 and 2021 was South Sudan (0.49%), whereas from 2021 to 2050, the Lao People’s Democratic Republic is projected to have the highest EAPC (0.60%) (Figure 4G–I and Supplemental Table 4).

### APC analysis on ID incidence, prevalence, and DALYs

**FIGURE 5:**
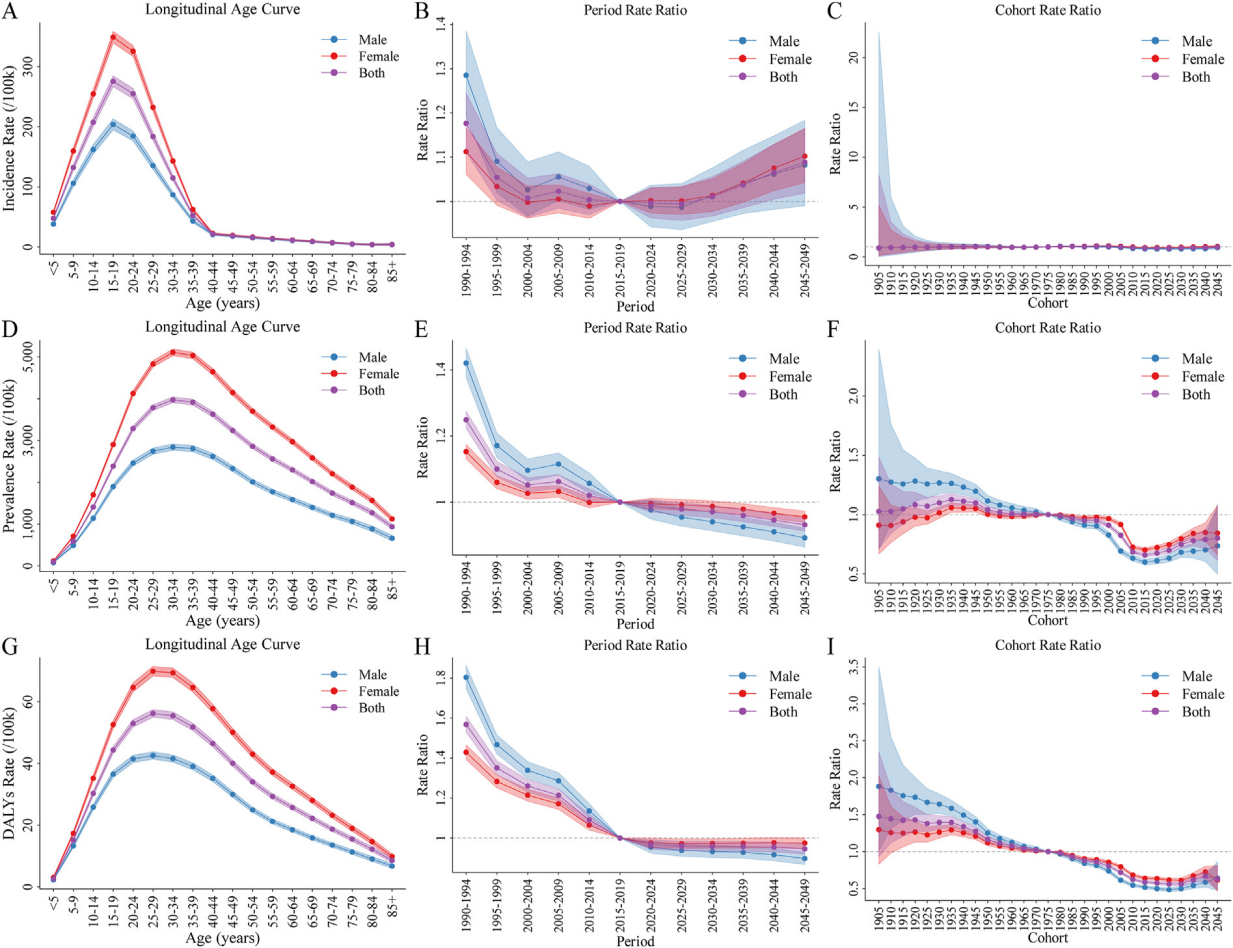
Age, period, and birth cohort effects for global ID incidence, prevalence, and DALY rate. (A–C) The age (A), period (B), and cohort (C) effects of incidence rate. (D–F) The age (D), period (E), and cohort (F) effects of prevalence rate. (G–I) The age (G), period (H), and cohort (I) effects of DALY rate. DALY, disability-adjusted life year; ID, iodine deficiency.

The APC model was used to analyze the age, period, and cohort effects on the incidence, prevalence, and DALYs of ID. Age effect results showed that incidence rates increased with age for individuals under 20, peaking in the 15–19-y age group (275.59/100,000). After age 20 y, incidence rates generally declined with age. Prevalence rate was highest in the 30–34-y age group (3973.92/100,000), increasing with age till 35 y, and then decreasing thereafter. The DALY rate peaked in the 25–29-y age group (56.22/100,000), rising rapidly before age 30 y and then declining with age (Figure 5A, D, G and Supplemental Table 5).

The period effect analysis revealed that the RRs for incidence, prevalence, and DALYs were highest during 1990–1994 compared with the reference group (2015–2019), with RRs of 1.18 (95% CI: 1.11, 1.24), 1.25 (95% CI: 1.22, 1.28), and 1.57 (95% CI: 1.53, 1.61), respectively (Supplemental Table 6, Figure 5B, E, H).

Birth cohort effect analysis revealed little variation in incidence cohort RR. Prevalence cohort RR remained stable before 2000, then showed a general decline after 2000. The DALYs cohort RR peaked for the 1905 cohort 1.48 (95% CI: 0.93, 2.35) and showed an overall downward trend thereafter (Supplemental Table 7, Figure 5C, F, I).

### SHAP analysis on the variables influencing ID incidence, prevalence, and DALY rates

SHAP values quantify the contribution of each feature to a model’s prediction, where the magnitude reflects the importance of the feature and the sign indicates the direction of its influence. A positive SHAP value suggests the feature increases the predicted outcome, whereas a negative value suggests it decreases the outcome. Mean absolute SHAP value is used to assess the overall importance of variables by capturing both positive and negative impacts.

#### SHAP analysis from the original XGBoost model

**FIGURE 6:**
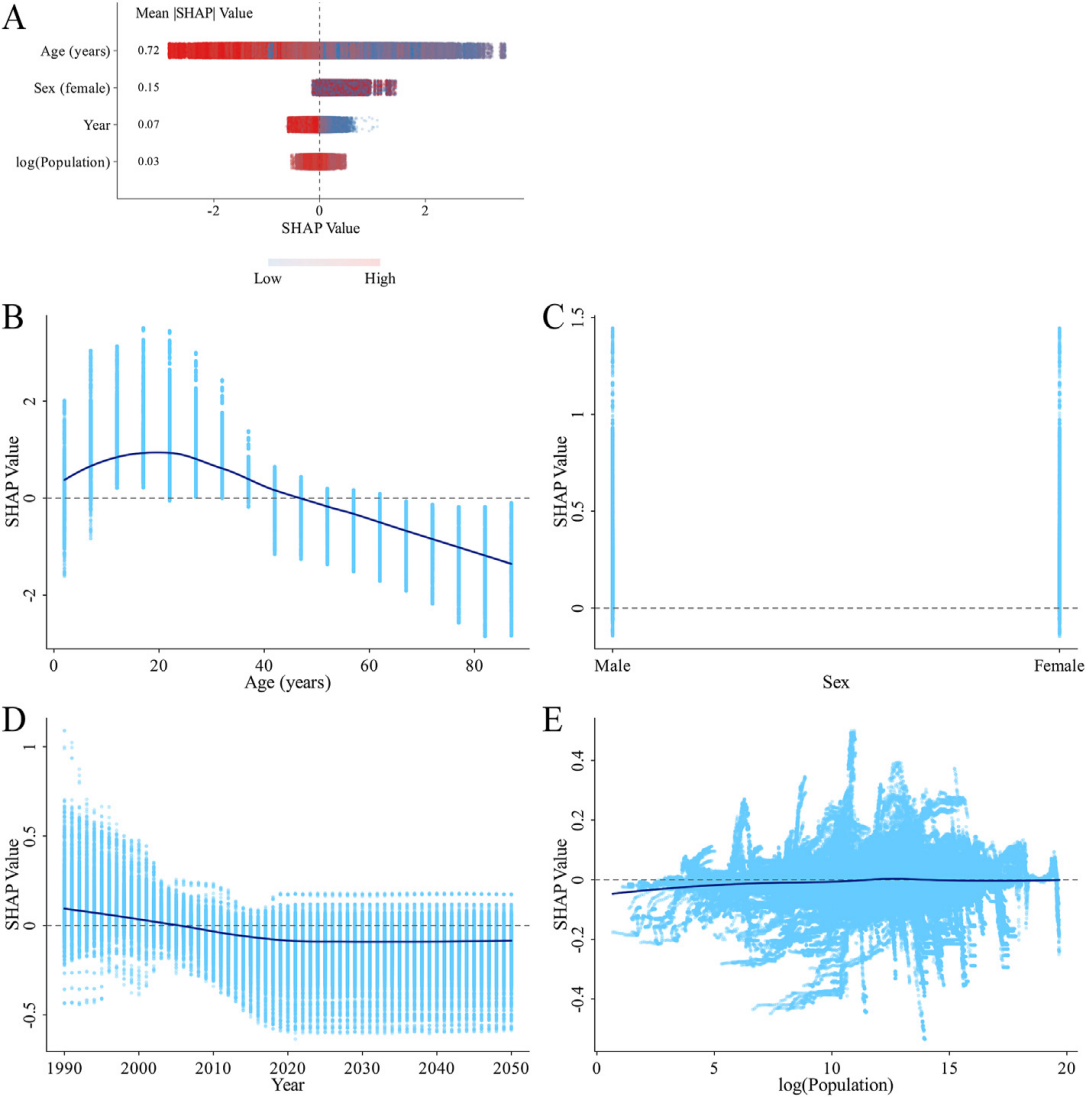
SHAP summary plot and dependence plots for features in the original XGBoost model predicting the ID incidence rate. (A) Summary plot. (B–E) The dependence plot showing the contribution of age (B), gender (C), year (D), and log (population) (E). ID, iodine deficiency; SHAP, Shapley additive explanations.

**FIGURE 7:**
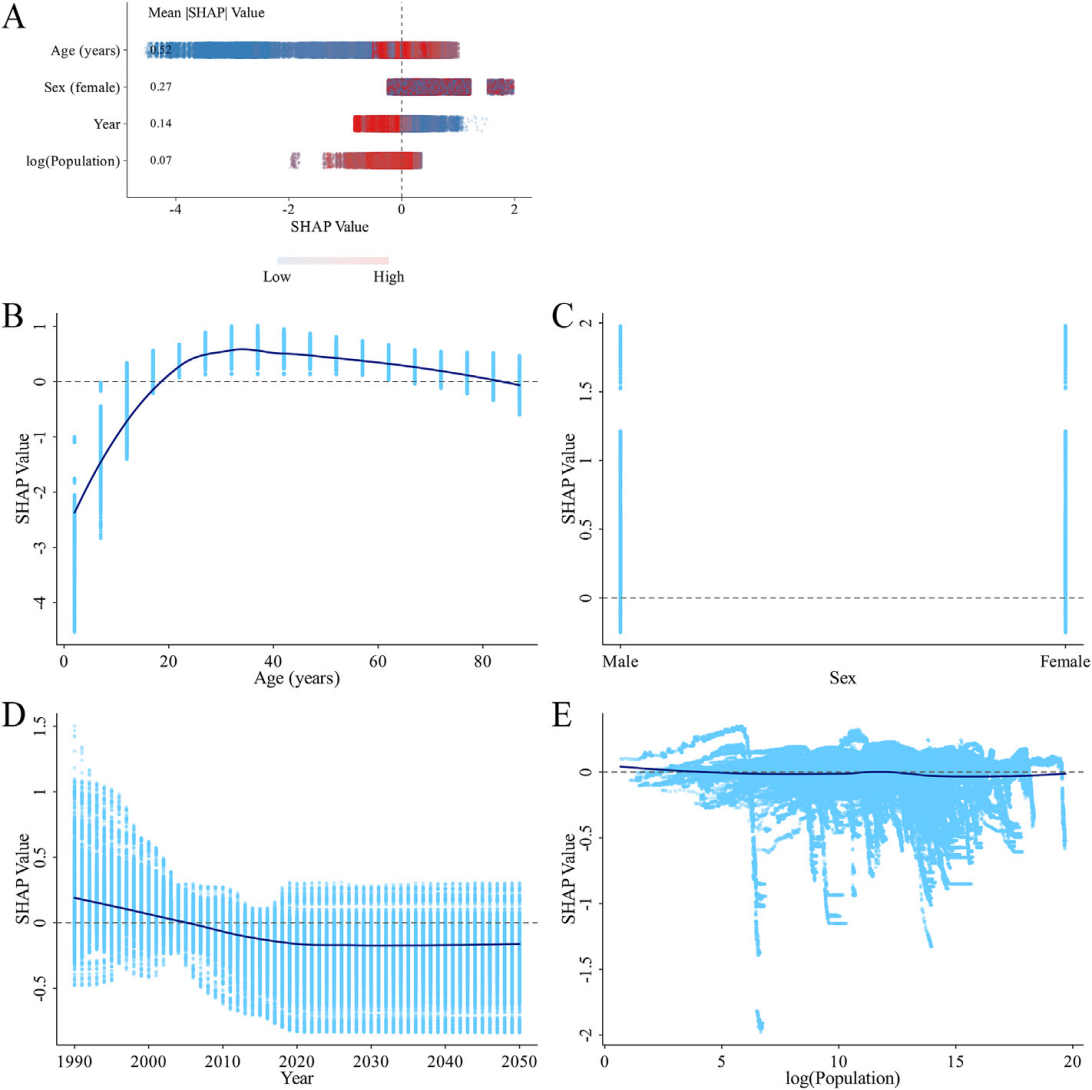
SHAP summary plot and dependence plots for features in the original XGBoost model predicting the ID prevalence rate. (A) Summary plot. (B–E) The dependence plot showing the contribution of age (B), gender (C), year (D), and log (population) (E). ID, iodine deficiency; SHAP, Shapley additive explanations.

**FIGURE 8:**
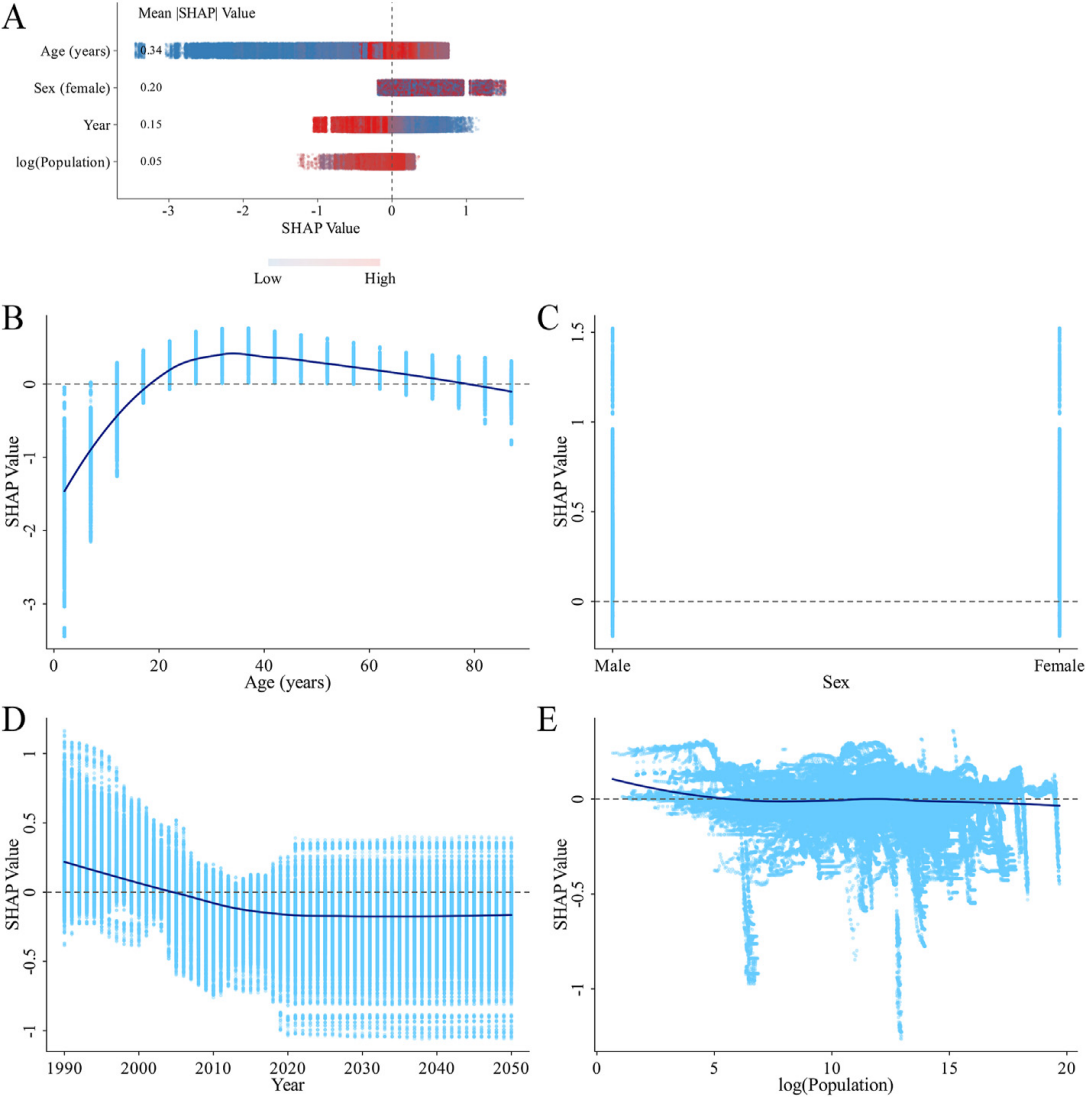
SHAP summary plot and dependence plots for features in the original XGBoost model predicting the ID DALY rate. (A) Summary plot. (B–E) The dependence plot showing the contribution of age (B), gender (C), year (D), and log (population) (E). DALY, disability-adjusted life year; ID, iodine deficiency; SHAP, Shapley additive explanations.

In the original XGBoost model, age, gender, and year were identified as the most influential factors on incidence, prevalence, and DALYs. The SHAP analysis showed that the impact of age, gender, and year on incidence, prevalence, and DALYs followed distinct patterns. Before age 20 y, age was generally positively correlated with incidence, but this correlation turned negative after 20 y. For prevalence and DALYs, the pattern was similar: before age 30 y, age was positively correlated, whereas after 30 y, the correlation became negative. SHAP values for gender indicated that females are at higher risk of incidence, prevalence, and DALYs than males. Year also showed a similar trend across all metrics: from 1990 to 2015, it was generally negatively correlated with incidence, prevalence, and DALYs, and the impact stabilized after 2015 (FIGURE 6, FIGURE 7, FIGURE 8).

When analyzing specific countries in 2021, the Democratic Republic of the Congo, Djibouti, and Somalia had the highest ASIRs, ASPRs, and ASDRs. SHAP analysis of incidence, prevalence, and DALYs in these countries showed similar patterns for age and year. For instance, in Djibouti and Somalia, age was positively correlated with incidence before age 20 y and negatively correlated afterward, whereas in the Democratic Republic of the Congo, this shift occurred around age 15 y. The impact of age on prevalence and DALYs was consistent across these countries, with positive correlations before age 30–35 y and negative correlations afterward (Supplemental Figures 4–12).

#### SHAP results from the iodized salt coverage-based XGBoost model

In the iodized salt coverage-based model, iodized salt coverage emerged as a critical predictor influencing ID burden across incidence, prevalence, and DALYs. The SHAP analysis revealed a distinct nonlinear relationship between iodized salt coverage and ID burden in most countries with available data. Specifically, when iodized salt coverage reaches a certain threshold, further increases are generally associated with a decline in incidence, prevalence, and DALY rates. However, the specific threshold varies across countries, reflecting regional differences in the impact of salt fortification programs. For example, among the 66 countries with available iodized salt coverage data, the 3 countries with the highest ASPRs in 2021 (Congo, Democratic Republic of the Congo, and Ethiopia) exhibited varying iodized salt coverage thresholds. In Congo, incidence, prevalence, and DALY rates began to decline significantly once iodized salt coverage exceeded 87%. In the Democratic Republic of the Congo, this threshold was slightly lower at 77%, whereas in Ethiopia, reductions in all 3 metrics were observed only after iodized salt coverage surpassed 50% (Supplemental Figure 13). These patterns indicated that adequate iodized salt coverage is a critical factor in reducing ID burden, but the effective coverage threshold varies by country. This underscores the importance of tailoring salt fortification programs to regional needs and ensuring sufficient coverage to achieve maximum health benefits.

Overall, SHAP values quantified the significant contribution of iodized salt coverage to reducing ID burden, alongside traditional predictors like age and sex. This analysis provided valuable insights into how scaling up salt iodization programs could impact ID burden in countries with available data, emphasizing its role as a critical intervention for public health improvement (Supplemental Figure 13).

### SDI and iodized salt coverage compared with ASRs of ID

**FIGURE 9:**
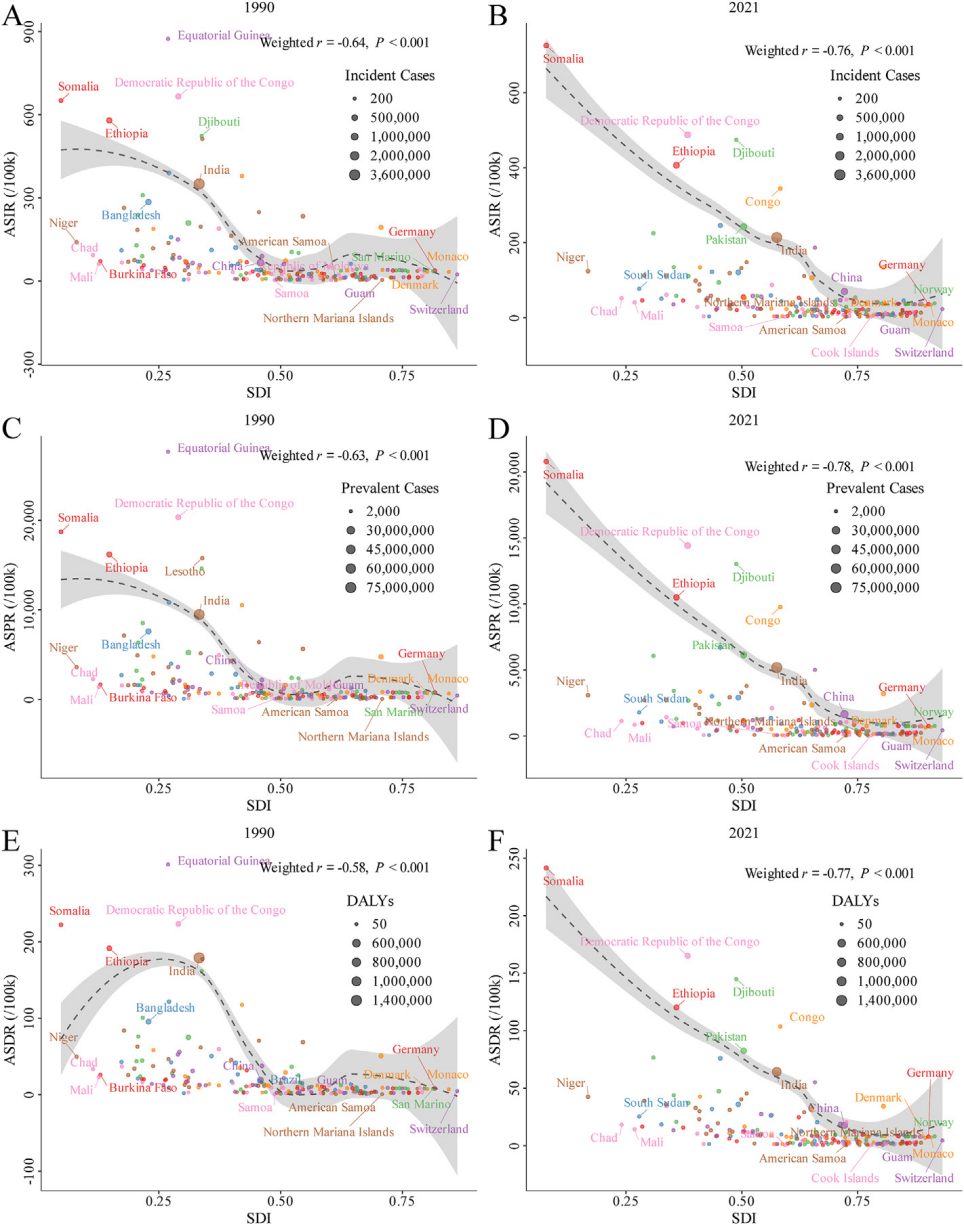
Weighted correlation analysis between the SDI and ID ASIR, ASPR, and ASDR at global and regional levels in 1990, 2021, and 2050, with incident case, prevalent case, and DALYs as weights. (A, B) ASIR in 1990 (A) and 2021 (B); (C, D) ASPR in 1990 (C) and 2021 (D); (E, F) ASDR in 1990 (E) and 2021 (F). ASDR, age-standardized DALY rate; ASIR, age-standardized incidence rate; ASPR, age-standardized prevalence rate; ASR, age-standardized rate; DALYs, disability-adjusted life year; ID, iodine deficiency; SDI, sociodemographic index.

SDI correlated negatively with ASIR, ASPR, and ASDR. The weighted correlation coefficients in 1990 were −0.64 (*P* < 0.01) for ASIR, −0.63 (*P* < 0.01) for ASPR, and −0.58 (*P* < 0.01) for ASDR. In 2021, the weighted correlation coefficients between SDI and ASIR, ASPR, and ASDR were −0.76 (*P* < 0.01), −0.78 (*P* < 0.01), and −0.77 (*P* < 0.01), respectively (Figure 9). These results suggested that higher socioeconomic development is associated with lower ASRs of ID burden.

For the 66 countries with available iodized salt coverage data, iodized salt coverage was also negatively correlated with ASIR, ASPR, and ASDR. In 1990, the weighted correlation coefficients were −0.49 (*P* < 0.01) for ASIR, −0.47 (*P* < 0.01) for ASPR, and −0.44 (*P* < 0.01) for ASDR. By 2021, these correlations weakened substantially, with coefficients of −0.04 (*P* = 0.741) for ASIR, −0.13 (*P* = 0.307) for ASPR, and −0.07 (*P* = 0.562) for ASDR (Supplemental Figure 14).

## Discussion

This study provided a detailed analysis of global and regional trends in ID using GBD 2021 data and predictive modeling with XGBoost and SHAP. The results from 1990 to 2021 showed a general decline in ID, but regions like Central Sub-Saharan Africa, South Asia, and Eastern Sub-Saharan Africa continue to experience high burdens, particularly among females and younger age groups. The prediction for 2022 to 2050 indicated that although the global ASIR, ASPR, and ASDR are expected to stabilize or slightly decrease, these 3 regions will continue to face significant challenges. Central Sub-Saharan Africa, South Asia, and Eastern Sub-Saharan Africa are projected to maintain the highest ASIR, ASPR, and ASDR of ID.

SHAP analysis highlighted age, sex, year, and iodized salt coverage as key factors influencing ID trends. Age showed a complex, shifting impact: for incidence, age correlated positively before 20 y and negatively correlated after. For prevalence and DALYs, age correlated positively until 30–35 y, after which the correlation turned negative. Gender analysis consistently showed higher risks for females in all metrics, although year showed a general negative correlation with ID from 1990 to 2015, with the effect stabilizing after 2015. Reductions in ID burden were generally observed only after achieving region-specific coverage thresholds, highlighting the need for tailored fortification strategies to address regional disparities.

These projections underscore the ongoing burden of ID in these vulnerable regions through 2050, emphasizing the need for sustained public health interventions. The use of XGBoost with SHAP enhances both the accuracy and interpretability of predictions, offering valuable insights for guiding future prevention strategies.

This study analyzed the global burden of ID and found significant variation across regions and over time. When interpreting the results, it is important to note the differences in ASIR estimates between the GBD 2021 and GBD 2019 studies. For instance, the 2019 ASIR of ID reported by GBD 2021 was 106.33 per 100,000, whereas the 2019 ASIR from GBD 2019 was 108.32 per 100,000. These 2 estimates are very close, reflecting a high degree of consistency between the 2 iterations. The small difference is primarily attributable to updates in the standard population structure used for age-standardization in GBD 2021, which reflects changes in global demographic trends. Importantly, the data sources and modeling strategies used to estimate the ASIR for 2019 were consistent between GBD 2019 and GBD 2021 [17,24]. Therefore, the observed differences in ASIR estimates are solely due to the revised standardization approach.

This highlights the need for careful consideration of adjustments in age-standardization when comparing results across GBD iterations. Although these updates improve the comparability and representativeness of the estimates, they may introduce slight differences when comparing metrics like ASIR between iterations. Such nuances are critical to ensuring an accurate interpretation of findings, particularly in studies analyzing trends over time.

The reliance on clinical outcomes, particularly visible goiter (grade 2), in the GBD framework provides a robust basis for assessing ID’s measurable health burden. However, the case definition does not incorporate stratification by age or biochemical markers, such as urinary iodine concentrations, which are often used in public health to identify subclinical ID. This limits the framework’s ability to capture early stages of deficiency or mild cases. Our study stratified results by age to provide a more granular understanding of ID trends. However, the GBD methodology itself does not explicitly support such stratification at the case definition level. Future research should aim to refine definitions and include biochemical and demographic stratifications to better capture the full spectrum of ID.

### Gender and age distribution in ID

The distribution of ID varies significantly across gender and age groups, influenced by physiologic demands, autoimmune tendencies, and the long-term effects of deficiency. Globally, women are disproportionately affected by ID, particularly during their reproductive years, due to increased iodine requirements during pregnancy and lactation. During pregnancy, iodine is critical for the production of thyroid hormones needed for both fetal neurodevelopment and maternal metabolism. Insufficient iodine intake during this period not only increases risk of maternal hypothyroidism and goiter but also leads to adverse fetal outcomes such as cretinism, impaired growth, and reduced cognitive abilities in children [25]. These findings align with global evidence, where iodine supplementation in pregnancy has been shown to significantly reduce such risks [26]. Furthermore, lactation increases iodine demands, further exacerbating the deficiency in regions with marginal iodine intake [27].

Women continue to exhibit a higher prevalence of ID-related diseases than men throughout their lives. Beyond reproductive years, women face sustained thyroid hormone demands due to chronic thyroid conditions, such as goiter or hypothyroidism, which may have developed during their reproductive years. These conditions can persist and worsen in the absence of adequate iodine intake, contributing to long-term health burdens [28]. Women are also more prone to autoimmune thyroid diseases, such as Hashimoto thyroiditis and Graves disease, which are exacerbated in iodine-deficient environments, increasing the burden of thyroid dysfunction [29]. Notably, even in iodine-sufficient regions, women exhibit higher rates of thyroid autoimmunity than men, likely due to hormonal influences, such as fluctuations in estrogen levels, and genetic predisposition [30]. These combined factors—higher iodine demands during key life stages such as pregnancy, lactation, and menopause, alongside a heightened susceptibility to thyroid autoimmunity and the long-term effects of chronic thyroid dysfunction—underscore the chronic vulnerability of women to ID and its associated complications. Addressing these unique challenges requires targeted public health interventions, including the implementation of iodine supplementation programs, improved access to iodized salt, and regular monitoring of iodine status, particularly in regions with insufficient or marginal iodine intake. These strategies are essential to mitigating the multifaceted burden of ID and improving health outcomes for women across their lifespan.

Younger populations, particularly those aged 10–30 y, show higher incidence due to increased thyroid hormone needs during growth and development. ID becomes more apparent after 5 y, with prevalence and DALYs peaking around 20–45 y. This is largely due to the cumulative effects of early ID, which can lead to chronic conditions such as goiter or hypothyroidism that persist throughout adulthood. Although the incidence of new ID cases tends to decline after 30 y, these chronic conditions continue to drive high prevalence and DALYs [31].

After age 35 or 40 y, ID prevalence and DALYs remain elevated, largely due to the chronic nature of thyroid disorders that develop earlier in life. Conditions such as goiter, hypothyroidism, and thyroid nodules, once established, often become permanent even if iodine intake improves later. These thyroid conditions can result in persistent morbidity, maintaining high levels of prevalence and DALYs in older age groups. Moreover, as individuals age, the thyroid gland’s ability to adapt to ID diminishes, leading to increased risk of complications. Additionally, older adults, particularly women, are at greater risk of autoimmune thyroid diseases such as Hashimoto thyroiditis, which further increases the disease burden in this age group [32].

To address these persistent patterns, targeted public health interventions are essential. Ensuring adequate iodine intake, particularly in women of childbearing age, children, and adolescents, should be a priority. Regular monitoring and iodine supplementation programs can help mitigate the long-term impacts of deficiency. Additionally, sustained iodine fortification efforts are crucial to reduce the prevalence of ID across all age groups, particularly in regions where the condition remains prevalent.

### SDI and ID in high-burden regions

The SDI is closely linked to the persistence of ID in regions like Central Sub-Saharan Africa, South Asia, and Eastern Sub-Saharan Africa, which experience the highest burdens. These regions, characterized by low SDI, are affected by poor socioeconomic conditions, limited education, and inadequate health care, all of which hinder efforts to address ID.

In Central and Eastern Sub-Saharan Africa, ID is driven by poor access to iodized salt and limited dietary diversity. Rural populations, in particular, rely on noniodized salt, and the infrastructure to support consistent iodization remains weak. As a result, conditions like goiter are common, especially among women and children, and low education and income levels further reduce the adoption of iodized salt [33].

In South Asia, despite efforts to implement universal salt iodization, coverage remains inconsistent due to political and logistical challenges. Large rural populations, combined with widespread poverty and malnutrition, particularly among women and children, exacerbate ID. This deficiency contributes to developmental delays and cognitive impairment, especially in areas with low SDI where public health interventions are uneven [34].

The low SDI in these regions correlates with poor awareness and access to iodized salt. Households with lower education and income are less likely to use iodized salt, perpetuating ID across generations [35].

Addressing ID in these regions will require expanding access to iodized salt, improving public education, and tackling broader socioeconomic challenges. These efforts can help reduce the burden of ID and its long-term health impacts on vulnerable populations.

### Feasibility and reliability of SHAP in analyzing ID

The use of SHAP in analyzing ID trends demonstrate strong feasibility, particularly given its consistency with findings from the APC model. Both SHAP and APC demonstrate similar patterns regarding age-related effects on incidence, prevalence, and DALYs, with incidence positively correlated with age before 20 y and declining thereafter. This consistency reinforces SHAP’s reliability in identifying key factors influencing ID, further confirming its suitability for use in health data analysis.

SHAP’s interpretability adds to its practicality, especially in public health decision-making. Unlike traditional machine learning models, which often act as black boxes, SHAP clearly explains the contributions of each factor, such as age, gender, or year to health outcomes. This feature makes SHAP particularly useful for public health professionals to identify high-risk groups and implement targeted interventions. For example, SHAP has been successfully used in other health studies to explain complex interactions, as shown in the work by Orsini et al. [36], where SHAP effectively revealed interaction effects between dietary and lifestyle factors on mortality outcomes. Such interpretability is key to its feasibility in health research.

SHAP’s feasibility can be further enhanced by integrating additional data sources, such as socioeconomic conditions, health care access, and environmental factors. Incorporating these variables would provide a more comprehensive understanding of ID’s underlying causes, particularly in regions where access to iodized salt or health care infrastructure is limited.

In summary, SHAP is a highly feasible tool for analyzing ID trends. Its interpretability and potential for integrating broader data make it a valuable resource for designing targeted public health interventions and addressing the multifaceted drivers of ID.

### Strengths and challenges of the XGBoost+SHAP model

The XGBoost+SHAP model applied in this study demonstrates several strengths. It offers high predictive accuracy, successfully forecasting ID trends across incidence, prevalence, and DALYs. This capability is vital for public health planning, particularly in regions like Central Sub-Saharan Africa and South Asia, where ID is still a major concern. Additionally, SHAP improves interpretability by clearly showing how variables such as age, gender, and year impact ID outcomes. This level of transparency is essential for identifying high-risk groups and shaping targeted interventions.

However, the model also faces several challenges. A major limitation is its narrow feature set, which includes only age, gender, year, and population size. This restricts the model’s ability to capture other important factors, such as dietary habits, health care access, and environmental influences, which can play a critical role in shaping ID trends. The model’s generalizability might also be constrained by these limitations. Although it performs well in broader analyses, its accuracy may decrease in regions with unique local factors that are not fully accounted for by the current input variables. Consequently, predictions in some areas may lack precision, especially where ID is influenced by factors beyond the scope of the model’s inputs. To address these challenges, expanding the feature set to include a broader range of data—such as socioeconomic or health care factors—could further enhance the model’s accuracy and applicability across different regions.

## Conclusions

This study provides a detailed analysis of global ID trends from 1990 to 2021, with projections through 2050. The use of the XGBoost+SHAP model effectively identified key factors such as age, gender, and year, offering accurate and interpretable predictions that are crucial for understanding regional and demographic disparities in ID. Although the model performs well, future enhancements could incorporate additional variables, such as socioeconomic factors, to improve its applicability in regions with unique local conditions. Despite global progress in reducing ID, regions like Central Sub-Saharan Africa, South Asia, and Eastern Sub-Saharan Africa remain heavily affected, particularly among women and younger populations. This underscores the need for continued, targeted public health interventions.

To address these challenges, expanding universal salt iodization programs should be a priority. Strengthening supply chains, ensuring access to iodized salt in rural and underserved regions, and enhancing quality control measures are critical. Public health campaigns should focus on educating women of reproductive age about the importance of iodine. In regions where iodized salt is insufficient, supplementation programs are essential. Routine monitoring of iodine levels in high-risk groups, particularly pregnant women and children, should be integrated into national health strategies. With coordinated international efforts, these measures can help mitigate the long-term health impacts of ID and improve outcomes for vulnerable populations.

## Author contributions

The authors’ responsibilities were as follows – LW, DL: performed the methodology, wrote the original draft, and reviews and edited the manuscript; LW: performed software analysis; DL, LW, PZ, JL, LC, QC, SL: performed formal analysis; ZL, CK, YL: administered the project; CK: was responsible for funding acquisition; and all authors: read and approved the final manuscript.

## Funding

This study was supported by the following: Guangdong Provincial Science and Technology Project (Guangdong Province, China; grant No. 2022B1111020005), National Key Research and Development Program of China (2023YFC3041600), and Guangdong Special Support Program (Leading Talents in Science and Technology Innovation; 2023A001, Contract Number: 0620220104).

## Data availability

The data sets generated for this study can be found in the GBD at https://vizhub.healthdata.org/gbd-results/ (accessed on 1 August, 2024).

## Conflict of interest

The authors report no conflicts of interest.
