# Supplementary material for: Global Burden of Iodine Deficiency: Insights and Projections to 2050 Using XGBoost and SHAP
Source: Adv Nutr. 2025 Feb 4;16(3):100384. doi: 10.1016/j.advnut.2025.100384 (PMC11909719; doi:10.1016/j.advnut.2025.100384)
Supplement: Multimedia component 2 [file mmc2.docx]

**Liang Dan, Wang Li et al. Global Burden of Iodine Deficiency: Insights and Projections to 2050 Using XGBoost and SHAP**

Table of Contents

[Supplemental Appendix 1. List of 66 countries and years with available iodized salt coverage data 3](#_Toc190961601)

[Supplemental Appendix 2. Calculation EAPC of ASR 5](#_Toc190961602)

[Supplemental Appendix 3. Detailed description of the XGBoost model 6](#_Toc190961603)

[Supplemental Appendix 4. Hyperparameter tuning through grid search and cross-validation 7](#_Toc190961604)

[Supplemental Appendix 5. Explaining iodine deficiency prediction using SHAP values 8](#_Toc190961605)

[Supplemental Appendix 6. APC model analysis of age, period, and birth cohort effects on global iodine deficiency incidence, prevalence, and DALYs 9](#_Toc190961606)

[Supplemental Figure 1. Evaluation of the XGBoost models’ predictive performance for ID incidence, prevalence, and DALYs rates 10](#_Toc190961607)

[Supplemental Figure 2. Male-to-female ratios of incidence, prevalence, and DALYs rates for iodine deficiency across age groups in 1990, 2021, and 2050 11](#_Toc190961608)

[Supplemental Figure 3. Number and rate of iodine deficiency from 1990 to 2050 at the global for incidence, prevalence and DALYs 12](#_Toc190961609)

[Supplemental Figure 4. SHAP summary plot and dependence plots for features in the original XGBoost model predicting the iodine deficiency incidence rate in Somalia 13](#_Toc190961610)

[Supplemental Figure 5. SHAP summary plot and dependence plots for features in the original XGBoost model predicting the iodine deficiency incidence rate in Democratic Republic of the Congo 14](#_Toc190961611)

[Supplemental Figure 6. SHAP summary plot and dependence plots for features in the original XGBoost model predicting the iodine deficiency incidence rate in Djibouti 15](#_Toc190961612)

[Supplemental Figure 7. SHAP summary plot and dependence plots for features in the original XGBoost model predicting the iodine deficiency prevalence rate in Somalia 16](#_Toc190961613)

[Supplemental Figure 8. SHAP summary plot and dependence plots for features in the original XGBoost model predicting the iodine deficiency prevalence rate in Democratic Republic of the Congo 17](#_Toc190961614)

[Supplemental Figure 9. SHAP summary plot and dependence plots for features in the original XGBoost model predicting the iodine deficiency prevalence rate in Djibouti 18](#_Toc190961615)

[Supplemental Figure 10. SHAP summary plot and dependence plots for features in the original XGBoost model predicting the iodine deficiency DALYs rate in Somalia 19](#_Toc190961616)

[Supplemental Figure 11. SHAP summary plot and dependence plots for features in the original XGBoost model predicting the iodine deficiency DALYs rate in Democratic Republic of the Congo 20](#_Toc190961617)

[Supplemental Figure 12. SHAP summary plot and dependence plots for features in the original XGBoost model predicting the iodine deficiency DALYs rate in Djibouti 21](#_Toc190961618)

[Supplemental Figure 13. SHAP dependence plots for features in the iodized salt coverage-based XGBoost model predicting the iodine deficiency incidence, prevalence, and DALYs rate in Congo, Democratic Republic of the Congo, and Ethiopia 22](#_Toc190961619)

[Supplemental Figure 14. Weighted correlation analysis between the iodized salt coverage and ID ASIR, ASPR, and ASDR at national levels in 1990, 2021, with incident case, prevalent case, and DALYs as weights 23](#_Toc190961620)

[Supplemental Movie 1. ASIR of ID at the national level from 1990 to 2050 24](#_Toc190961621)

[Supplemental Movie 2. ASPR of ID at the national level from 1990 to 2050 24](#_Toc190961622)

[Supplemental Movie 3. ASDR of ID at the national level from 1990 to 2050 24](#_Toc190961623)

**Liang Dan, Wang Li et al. Global Burden of Iodine Deficiency: Insights and Projections to 2050 Using XGBoost and SHAP**

### Supplemental Appendix 1. List of 66 countries and years with available iodized salt coverage data

| **Country** | **Available Years of Iodized Salt Coverage Data** |
| --- | --- |
| Afghanistan | 2004, 2011, 2015 |
| Albania | 2000, 2005, 2009, 2017 |
| Algeria | 2006, 2012, 2019 |
| Armenia | 2000, 2005, 2016 |
| Azerbaijan | 2000, 2006, 2011, 2013 |
| Bangladesh | 2004-2006, 2013, 2015, 2019 |
| Benin | 1996, 2001, 2006, 2011-2012, 2014, 2018 |
| Bolivia (Plurinational State of) | 1994, 2000, 2003, 2008, 2016 |
| Burkina Faso | 2003, 2006, 2010, 2021 |
| Burundi | 2000, 2010, 2016 |
| Cambodia | 2000, 2010-2011, 2014 |
| Cameroon | 1998, 2000, 2004, 2006, 2011, 2014, 2018 |
| Central African Republic | 1994, 2000, 2006, 2010, 2012, 2019 |
| Chad | 1997, 2000, 2004, 2010, 2019 |
| China | 2009-2011, 2014, 2018 |
| Congo | 2005, 2011, 2014 |
| Côte d'Ivoire | 2000, 2012, 2016 |
| Democratic People's Republic of Korea | 2009-2010, 2017 |
| Democratic Republic of the Congo | 2001, 2007, 2010, 2013, 2017 |
| Egypt | 2000, 2003, 2005, 2008 |
| Eswatini | 2000, 2006, 2010, 2014 |
| Ethiopia | 2000, 2005, 2011, 2015-2016 |
| Gambia | 2000, 2006, 2010, 2013, 2018, 2020 |
| Ghana | 1998, 2003, 2006, 2011, 2014-2015, 2017 |
| Guatemala | 1995, 1999, 2009 |
| Guinea | 1999, 2005, 2007, 2012, 2016, 2018 |
| Guinea-Bissau | 2000, 2006, 2010, 2012, 2014, 2019 |
| Haiti | 2000, 2006, 2012, 2017 |
| India | 1999, 2006, 2018, 2020 |
| Iraq | 2000, 2006, 2011, 2018 |
| Kazakhstan | 1999, 2006, 2010, 2015 |
| Kenya | 2000, 2008, 2014 |
| Kyrgyzstan | 2006, 2012, 2014, 2018 |
| Lao People's Democratic Republic | 2000, 2006, 2011, 2017 |
| Lesotho | 2000, 2004, 2009, 2014 |
| Liberia | 2011, 2013, 2019 |
| Libya | 2003, 2007, 2014 |
| Madagascar | 1997, 2000, 2004, 2009 |
| Malawi | 2000, 2006, 2010, 2014-2015, 2020 |
| Mali | 2001, 2006, 2010, 2015, 2018, 2020 |
| Mauritania | 2000, 2007, 2011, 2015, 2018 |
| Mongolia | 2000, 2005, 2010, 2013, 2016, 2018 |
| Mozambique | 2003, 2008, 2011 |
| Myanmar | 2000, 2016, 2018 |
| Nepal | 1998, 2005, 2011, 2014, 2016 |
| Niger | 1998, 2000, 2006, 2010, 2012, 2014 |
| Nigeria | 1999, 2003, 2007-2008, 2011, 2018 |
| Paraguay | 1996, 2011, 2019 |
| Peru | 2000, 2008, 2010-2020 |
| Philippines | 1999, 2013, 2018 |
| Republic of Moldova | 2000, 2005, 2012 |
| Rwanda | 2000, 2005, 2010, 2015 |
| Sao Tome and Principe | 2000, 2008, 2014, 2019 |
| Senegal | 2000, 2005, 2011, 2013-2018 |
| Sierra Leone | 2000, 2005, 2007-2008, 2010, 2013, 2017, 2019 |
| Sri Lanka | 2000, 2007, 2010, 2016 |
| Tajikistan | 2000, 2005, 2007, 2009, 2012, 2016-2017 |
| Thailand | 2006, 2012, 2016, 2019 |
| Timor-Leste | 2007, 2013, 2016 |
| Togo | 2006, 2010, 2014, 2017 |
| Trinidad and Tobago | 2000, 2006, 2011 |
| Turkmenistan | 2000, 2006, 2015 |
| Uganda | 1995, 2000, 2006, 2011, 2016 |
| United Republic of Tanzania | 1999, 2004, 2010-2011, 2013 |
| Zambia | 1996, 1999, 2002, 2013 |
| Zimbabwe | 2010, 2014-2015, 2019 |

The iodized salt coverage data for 66 countries were sourced from the World Health Organization’s (WHO) Nutrition Landscape Information System (NLIS) (https://www.who.int/data/nutrition/nlis/data-search)

**Liang Dan, Wang Li et al. Global Burden of Iodine Deficiency: Insights and Projections to 2050 Using XGBoost and SHAP**

### Supplemental Appendix 2. Calculation EAPC of ASR

The EAPC was calculated using a linear regression model of the form:

$$y= \beta_{0}+\beta_{1}*x_{1}$$

In this model, $x_{1}$ represents the calendar year, and $y$ is the natural logarithm of the ASR, which can denote various measures such as the ASIR, ASPR, and ASDR. $\beta_{0}$ is the intercept, and $\beta_{1}$ is the coefficient corresponding to the calendar year.

The EAPC reflects the annual trend in ASRs over a defined period (e.g., 1990-2021 or 2021-2050) and was derived using the following formula ^1^:

$$EAPC=100*(exp\left( \beta_{1} \right)-1)$$

Confidence intervals for the EAPCs were computed by applying the upper and lower bounds of $\beta_{1}$​ from the regression analysis. This measure provides valuable insights into the direction and magnitude of changes in disease burden over time, helping to quantify whether ASRs are increasing, decreasing, or remaining stable over specified periods.

**Abbreviations:**

EAPC, estimated annual percentage change

ASR, age-standardized rate

ASIR, age-standardized incidence rate

ASPR, age-standardized prevalence rate

ASDR, age-standardized DALYs rate

DALYs, disability-adjusted life years.

**Reference**

1. Hankey B F, Ries L A, Kosary C L, et al. Partitioning linear trends in age-adjusted rates[J]. Cancer Causes & Control, 2000, 11: 31-35.

**Liang Dan, Wang Li et al. Global Burden of Iodine Deficiency: Insights and Projections to 2050 Using XGBoost and SHAP**

### Supplemental Appendix 3. Detailed description of the XGBoost model

XGBoost, a machine learning algorithm, generates decision trees iteratively to make predictions. With each step, it refines the model by adding new trees aimed at reducing the prediction errors from the previous iteration. The model’s objective function consists of two key components: a loss function, which measures the difference between predicted and actual values, and a regularization term, which limits model complexity to avoid overfitting ^1^. The objective function can be represented as:

$$\mathcal{L}\left( \boldsymbol{\phi} \right)=\sum_{i=1}^{n} l\left( \hat{y}_{i}, y_{i} \right)+ \sum_{k=1}^{K} \Omega\left( f_{k} \right)$$

Where:

$\mathcal{L}\left( \boldsymbol{\phi} \right)$ is the overall objective function, combining the loss and regularization components.

$l(\hat{y}_{i}, y_{i})$ represents the loss function for each instance, measuring how closely the predicted value $\hat{y}_{i}$ matches the actual value $y_{i}$.

$\Omega\mathbf{(}f_{k})$ is the regularization term applied to each tree $f_{k}$, controlling the complexity of the model to ensure generalization to unseen data.

The regularization term can be expressed as:

$$\Omega\left( f_{k} \right)\mathbf{=}\gamma T\mathbf{+}\frac{1}{2}\lambda\sum_{j=1}^{T} w_{j}^{2}$$

Where:

$T$ is the number of leaf nodes in a tree, with more nodes representing a more complex tree.

$w_{j}$ denotes the weight assigned to the $j$-th leaf node, influencing its contribution to the model’s predictions.

$\gamma$ penalizes the number of leaf nodes, preventing trees from becoming too large and overfitting.

$\lambda$ controls the magnitude of the leaf weights, encouraging smaller weights to make the model more conservative and stable.

**Reference**

1. Chen T, Guestrin C. Xgboost: A scalable tree boosting system[C]. Proceedings of the 22nd acm sigkdd international conference on knowledge discovery and data mining. 2016: 785-794.

**Liang Dan, Wang Li et al. Global Burden of Iodine Deficiency: Insights and Projections to 2050 Using XGBoost and SHAP**

### Supplemental Appendix 4. Hyperparameter tuning through grid search and cross-validation

To optimize the XGBoost model’s performance and improve prediction accuracy, a grid search was conducted to fine-tune critical hyperparameters: learning rate (*eta*), maximum tree depth (*max_depth*), and the number of boosting rounds (*nrounds*). This process was guided by 5-fold cross-validation, with RMSE serving as the evaluation metric for each set of hyperparameter values.

The *max_depth* hyperparameter controls the complexity of the decision trees. Deeper trees capture more intricate patterns within the data but may increase the likelihood of overfitting, whereas shallower trees reduce this risk but may miss important nuances. Values between 5 and 12 were tested to determine the optimal tree complexity.

The *eta* hyperparameter, which adjusts the learning rate, influences the size of the steps taken during the model’s optimization process. Lower values result in more cautious learning, requiring more iterations to reach convergence, while higher values accelerate convergence but may lead to suboptimal solutions. The range of *eta* values explored included 0.01, 0.015, 0.025, 0.05, 0.1, 0.15, 0.2, 0.25, and 0.3.

For the *nrounds* hyperparameter, which determines the number of boosting rounds, a range of 100 to 1500 (in increments of 100) was tested. Too few boosting rounds can lead to underfitting, while too many can result in overfitting. This range was chosen to find the balance between sufficient learning and avoiding over-complexity.

Through systematic experimentation with these hyperparameters, the goal was to identify the optimal combination that maximizes model accuracy while minimizing errors in the predictions.

**Abbreviations:**

RMSE, root mean square error

**Liang Dan, Wang Li et al. Global Burden of Iodine Deficiency: Insights and Projections to 2050 Using XGBoost and SHAP**

### Supplemental Appendix 5. Explaining iodine deficiency prediction using SHAP values

SHAP values provided a clear understanding of how various factors, such as age, sex, and population size, influenced the model's predictions for iodine deficiency. By quantifying the impact of each feature, SHAP highlighted whether a factor increased or decreased the predicted risk, offering valuable insights into which factors played the most critical roles in the model’s predictions. This enhanced our ability to interpret the key drivers behind the model’s outcomes ^1^. The SHAP value for a given feature $j$ is calculated using the following formula:

$$\phi_{j}=\sum_{S\subseteq F\backslash\{j\}} \frac{\left| S \right|!\left( \left| F \right|-\left| S \right|-1 \right)!}{|F|!}[f\left( S\cup\left\{ j \right\} \right)-f(S)]$$

In this formula:

$\phi_{j}$ represents the contribution of feature $j$ to the prediction, based on its impact when included in different subsets of features.

$S\subseteq F\backslash\{j\}$ denotes all subsets of the full feature set, excluding feature $j$.

The term $\frac{\left| S \right|!\left( \left| F \right|-\left| S \right|-1 \right)!}{|F|!}$ assigns a weight to each subset based on its size, where:

$\left| S \right|!$ accounts for the possible arrangements of features within subset $S$.

(|F|-|S|-1)! considers the features not included in $S$, except for $j$.

$\left| F \right|!$ normalizes the weights across different subset sizes.

$f\left( S\cup\left\{ j \right\} \right)$ is the model’s prediction with feature $j$ included, $f(S)$ is the prediction without feature $j$.

By utilizing SHAP values, we were able to clearly identify the importance of each variable in predicting iodine deficiency metrics like incidence, prevalence or DALYs, offering a detailed view of how individual factors contributed to the overall model performance.

**Abbreviations:**

SHAP, SHapley Additive exPlanations

**Reference**

1. Lundberg S. A unified approach to interpreting model predictions[J]. arXiv preprint arXiv:1705.07874, 2017.

**Liang Dan, Wang Li et al. Global Burden of Iodine Deficiency: Insights and Projections to 2050 Using XGBoost and SHAP**

### Supplemental Appendix 6. APC model analysis of age, period, and birth cohort effects on global iodine deficiency incidence, prevalence, and DALYs

To analyze the age, period, and birth cohort effects on global iodine deficiency incidence, prevalence, and DALYs, we utilized data from GBD 2021, incorporating population counts and health outcomes. Given the challenge of collinearity between age, period, and cohort, the IE method was employed to enhance the accuracy of the model’s parameters^1^. The model required equal intervals for age and period groups, so data for individuals aged 0 to 85+ years were grouped into 18 five-year age brackets (e.g., <5, 5-9, ..., 85+).

For the years spanning 1990 to 2049, data were divided into 12 five-year periods (e.g., 1990-1994, 1995-1999, ..., 2045-2049). Birth cohorts were categorized into 29 groups, ranging from 1903-1907 (midpoint 1905) to 2043-2047 (midpoint 2045). For each period and cohort, averages were calculated for population size, incidence, prevalence, and DALYs. RRs were used to assess the impact of period and cohort on iodine deficiency outcomes, with an RR greater than 1 indicating higher risk compared to the reference group, and an RR below 1 suggesting lower risk.

**Abbreviations:**

APC, Age-period-cohort

DALYs, disability-adjusted life years

GBD, Global Burden of Disease

IE, intrinsic Estimator

RR, rate ratio

**Reference**

1. Yang, Yang., Sam, Schulhofer-Wohl., Wenjiang, J., Fu., Kenneth, C., Land. (2008). The intrinsic estimator for age-period-cohort analysis: What it is and how to use it. American Journal of Sociology, 113(6):1697-1736. doi: 10.1086/587154

**Liang Dan, Wang Li et al. Global Burden of Iodine Deficiency: Insights and Projections to 2050 Using XGBoost and SHAP**

### Supplemental Figure 1. Evaluation of the XGBoost models’ predictive performance for ID incidence, prevalence, and DALYs rates


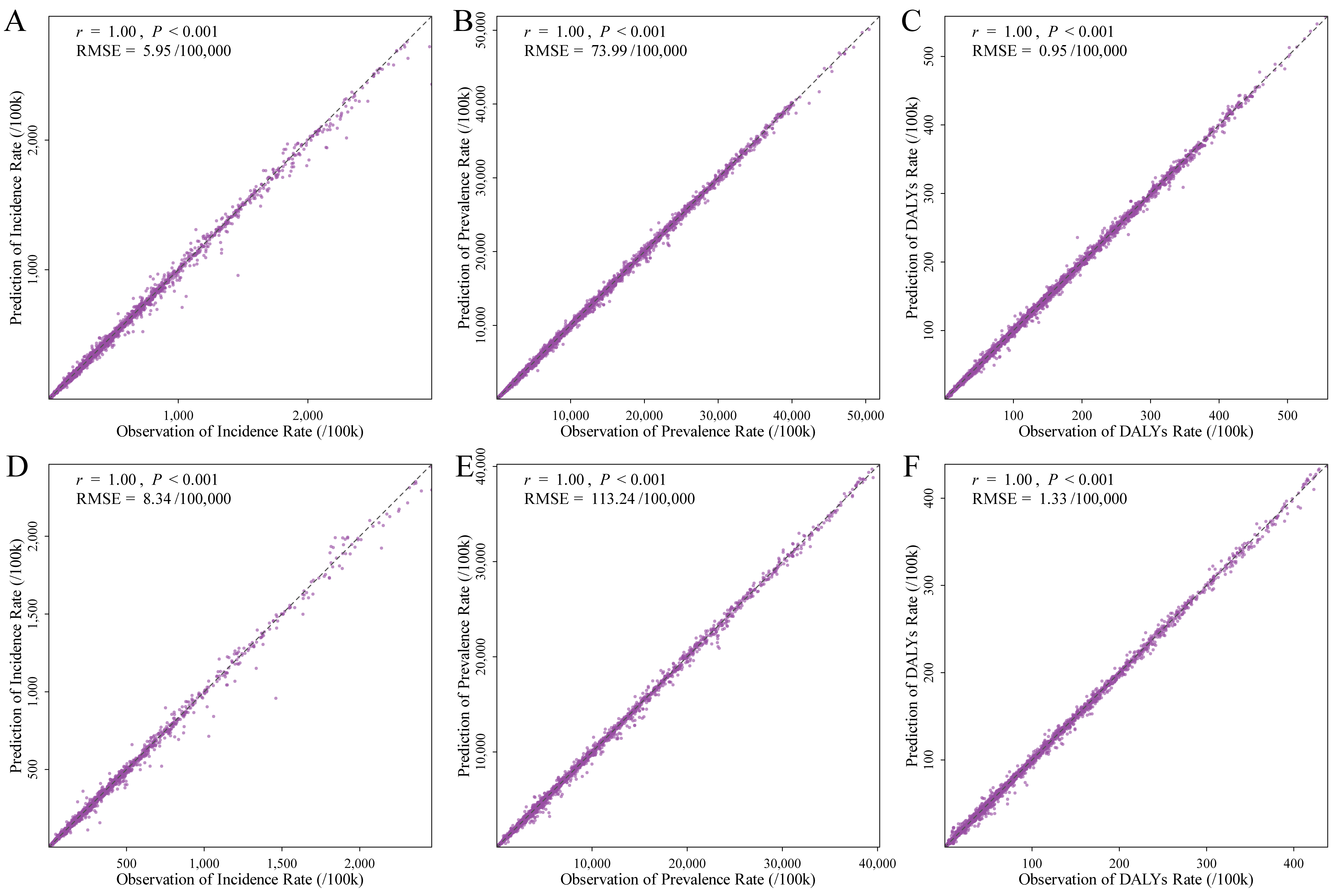


RMSE and Pearson correlation coefficients between observed and predicted values in incidence (A), prevalence (B), and DALYs (C) of ID for the original XGBoost model.

RMSE and Pearson correlation coefficients between observed and predicted values in incidence (D), prevalence (E), and DALYs (F) of ID for the iodized salt coverage-based XGBoost model.

**Abbreviations:**

ID, iodine deficiency

RMSE, root mean square error

DALY, disability-adjust life years

**Liang Dan, Wang Li et al. Global Burden of Iodine Deficiency: Insights and Projections to 2050 Using XGBoost and SHAP**

### Supplemental Figure 2. Male-to-female ratios of incidence, prevalence, and DALYs rates for iodine deficiency across age groups in 1990, 2021, and 2050


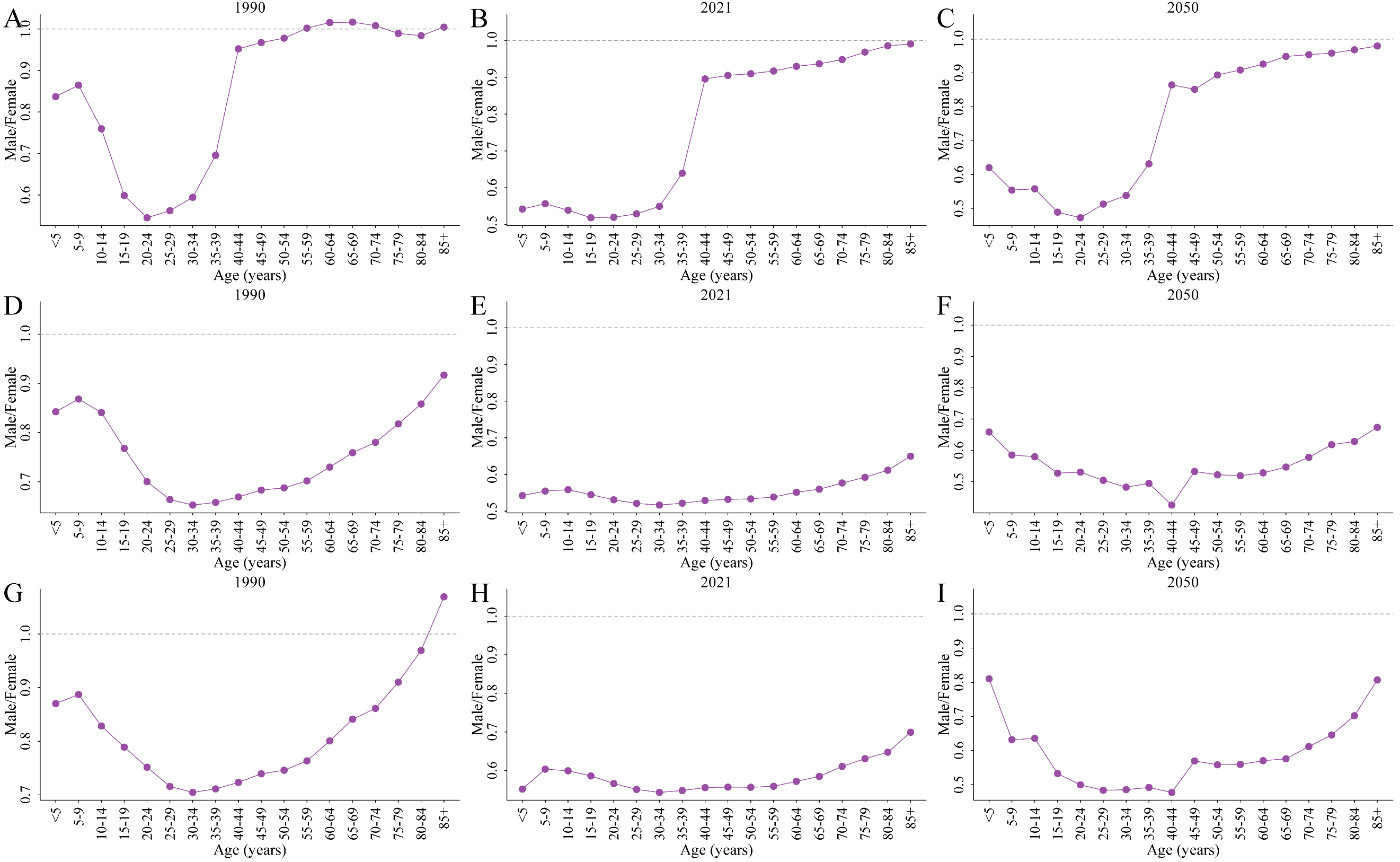


Incidence rate in 1990 (A), 2021 (B), and 2050 (C); Prevalence rate in 1990 (D), 2021 (E), and 2050 (F); DALYs rate in 1990 (G), 2021 (H), and 2050 (I).

**Abbreviations:**

DALYs, disability-adjusted life years

**Liang Dan, Wang Li et al. Global Burden of Iodine Deficiency: Insights and Projections to 2050 Using XGBoost and SHAP**

### Supplemental Figure 3. Number and rate of iodine deficiency from 1990 to 2050 at the global for incidence, prevalence and DALYs


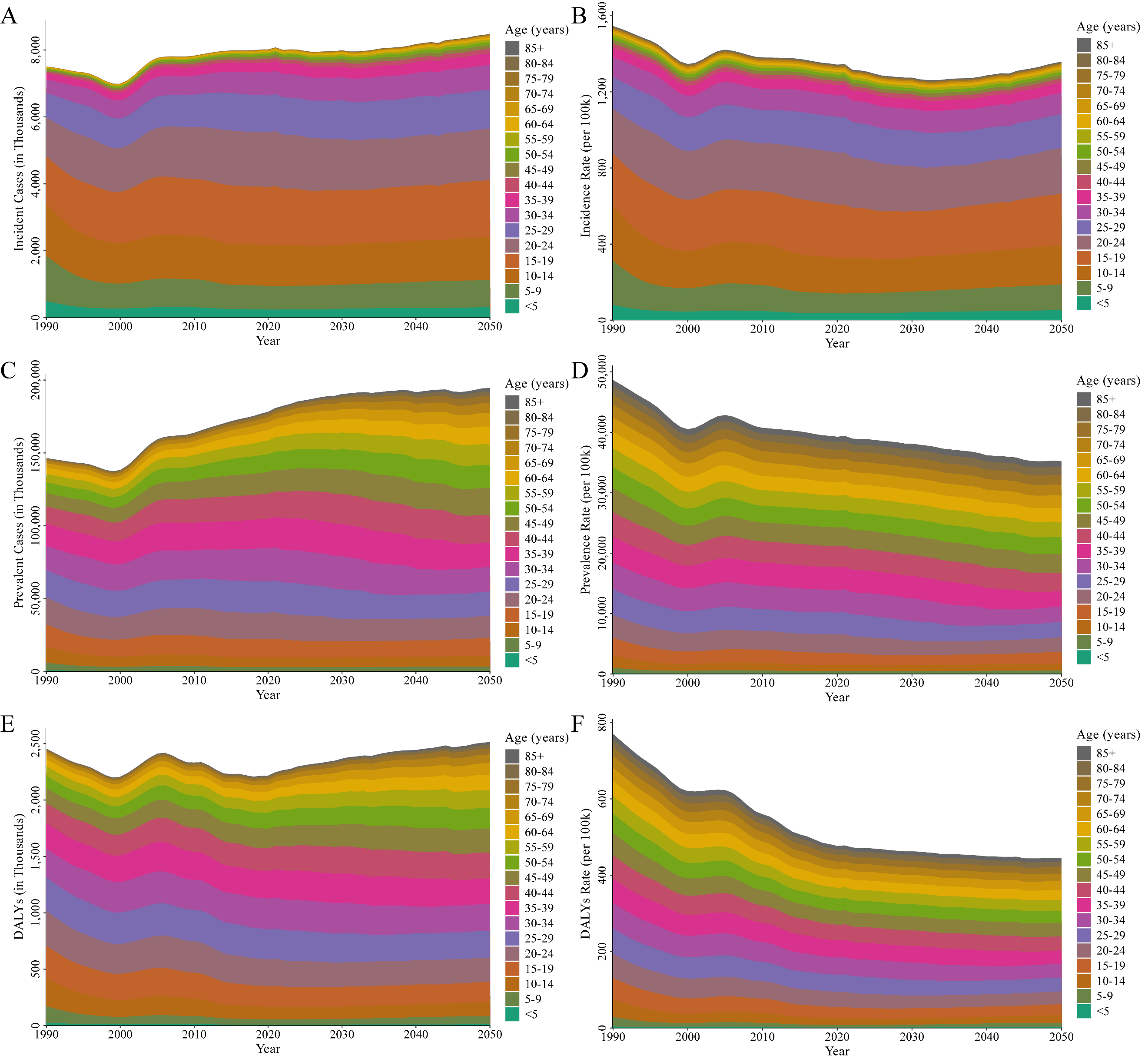


Incident cases (A); Incidence rate (B); Prevalent case (C); Prevalence rate (D); DALYs (E); DALYs rate (D).

**Abbreviations:**

DALYs, disability-adjusted life years

**Liang Dan, Wang Li et al. Global Burden of Iodine Deficiency: Insights and Projections to 2050 Using XGBoost and SHAP**

### Supplemental Figure 4. SHAP summary plot and dependence plots for features in the original XGBoost model predicting the iodine deficiency incidence rate in Somalia


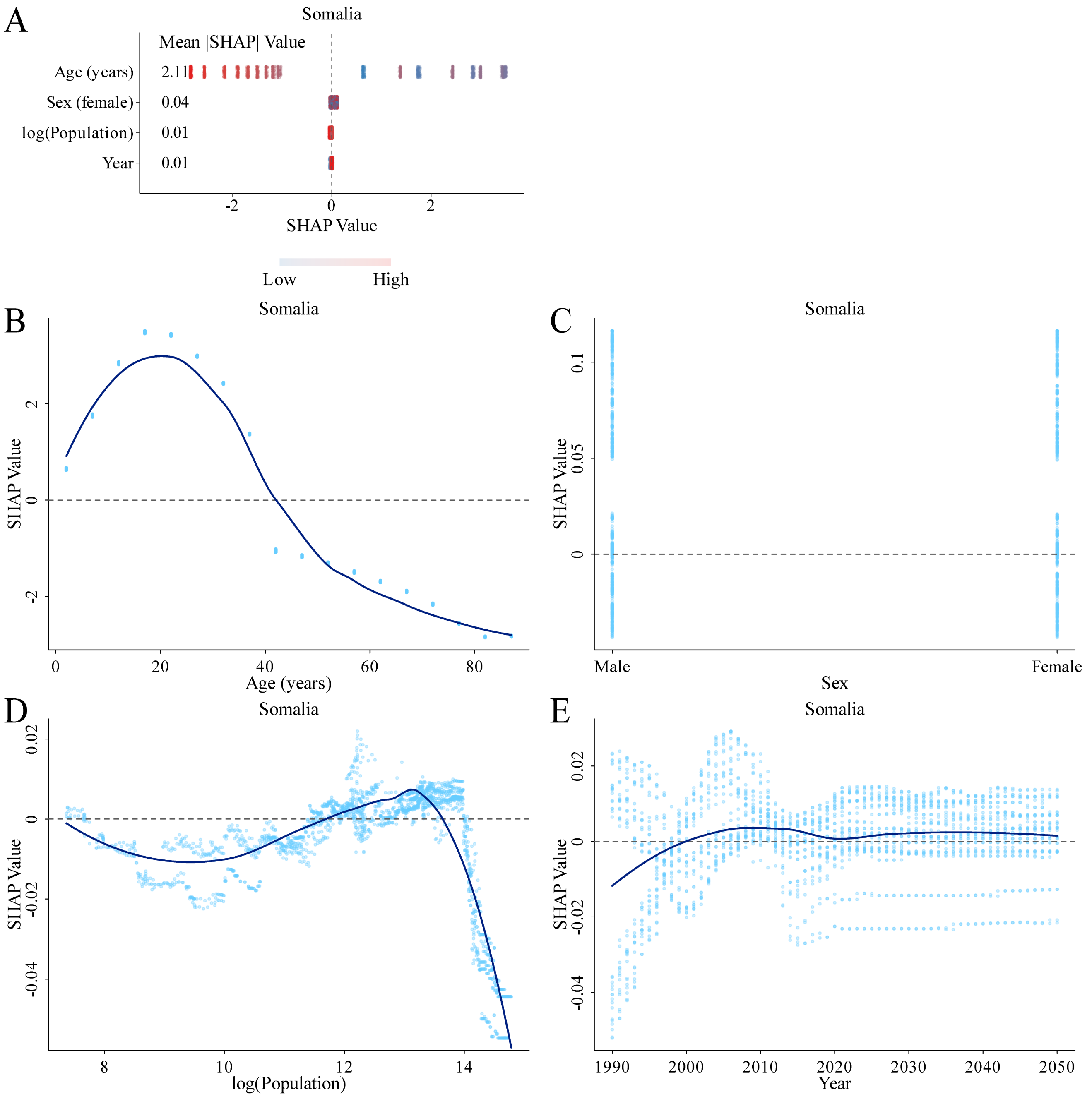


SHAP summary plot (A); Dependence plot of age (B), sex (C), log(population) (D), and year (E).

**Abbreviations:**

SHAP, SHapley Additive exPlanations

**Liang Dan, Wang Li et al. Global Burden of Iodine Deficiency: Insights and Projections to 2050 Using XGBoost and SHAP**

### Supplemental Figure 5. SHAP summary plot and dependence plots for features in the original XGBoost model predicting the iodine deficiency incidence rate in Democratic Republic of the Congo


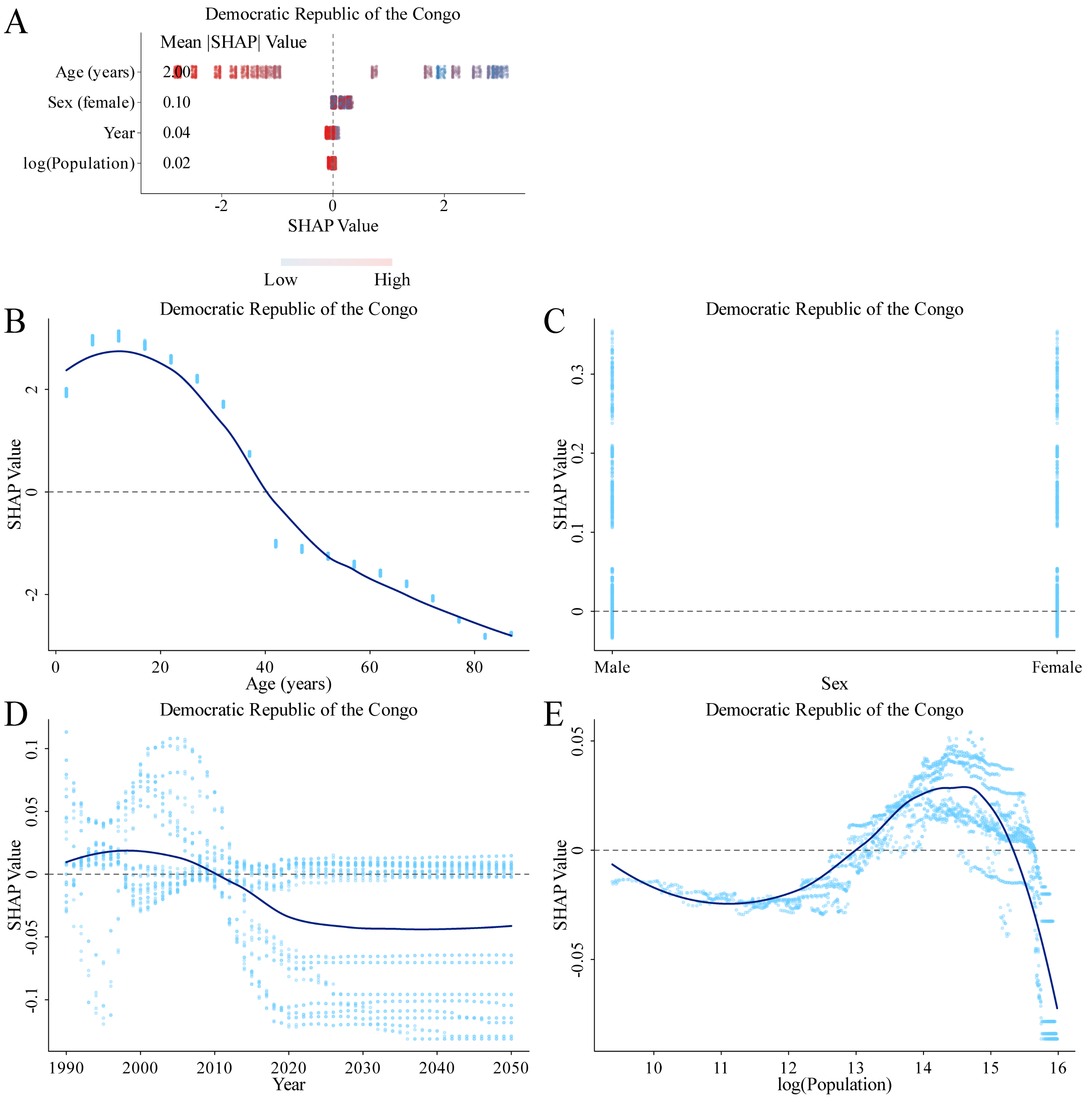


SHAP summary plot (A); Dependence plot of age (B), sex (C), year (D), and log(population) (E).

**Abbreviations:**

SHAP, SHapley Additive exPlanations

**Liang Dan, Wang Li et al. Global Burden of Iodine Deficiency: Insights and Projections to 2050 Using XGBoost and SHAP**

### Supplemental Figure 6. SHAP summary plot and dependence plots for features in the original XGBoost model predicting the iodine deficiency incidence rate in Djibouti


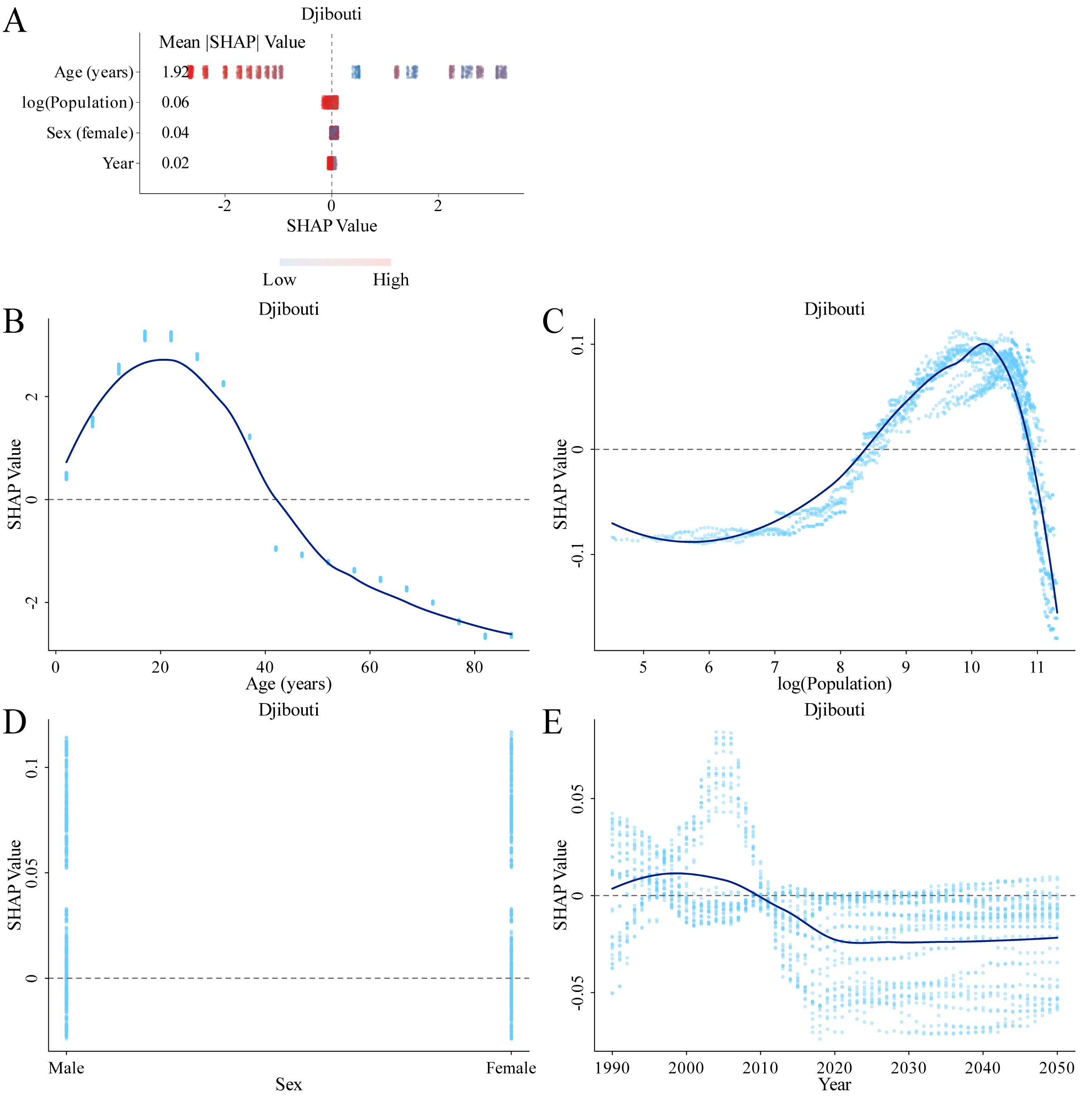


SHAP summary plot (A); Dependence plot of age (B), log(population) (C), sex (D), and year (E).

**Abbreviations:**

SHAP, SHapley Additive exPlanations

**Liang Dan, Wang Li et al. Global Burden of Iodine Deficiency: Insights and Projections to 2050 Using XGBoost and SHAP**

### Supplemental Figure 7. SHAP summary plot and dependence plots for features in the original XGBoost model predicting the iodine deficiency prevalence rate in Somalia


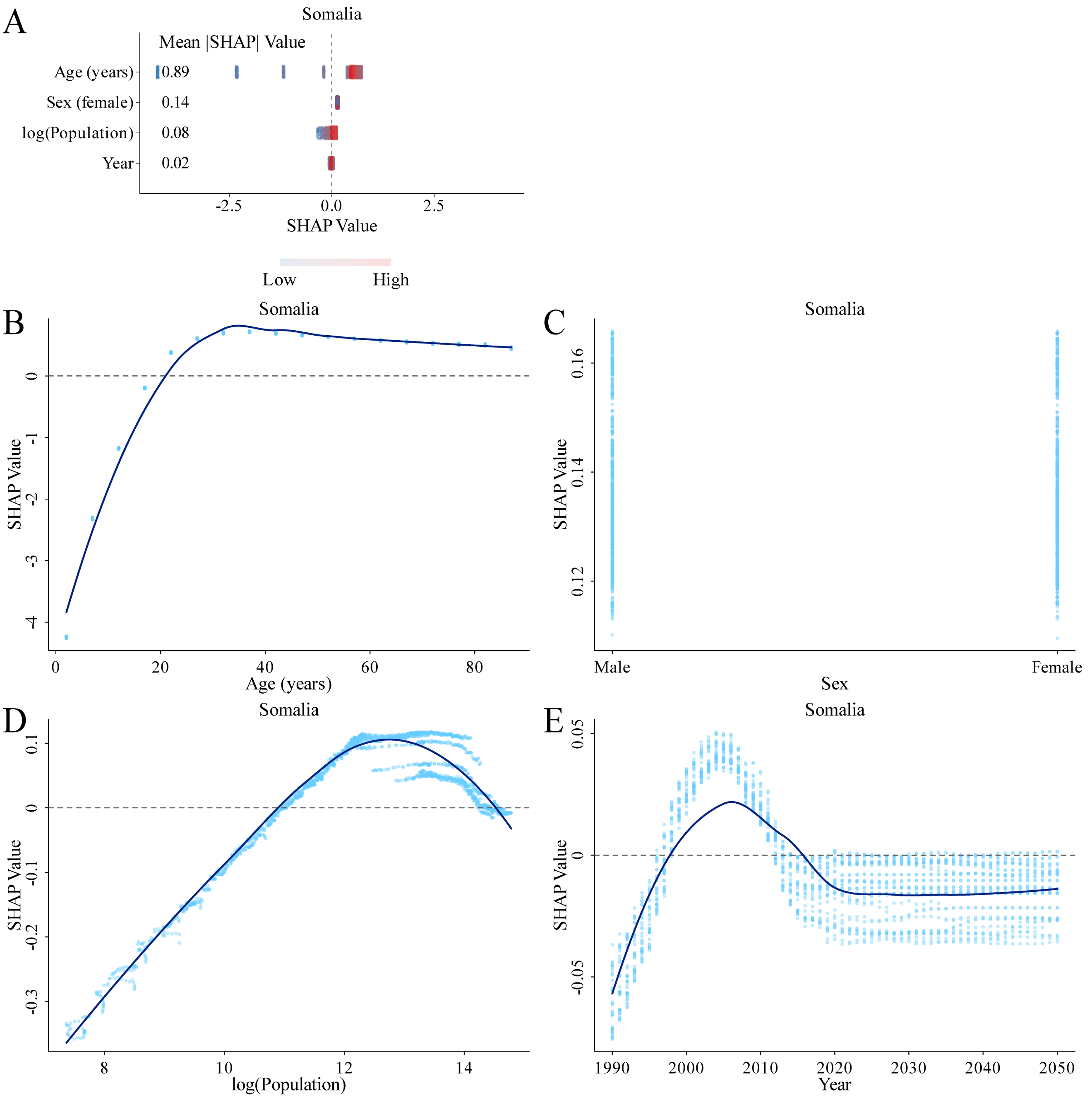


SHAP summary plot (A); Dependence plot of age (B), sex (C), log(population) (D), and year (E).

**Abbreviations:**

SHAP, SHapley Additive exPlanations

**Liang Dan, Wang Li et al. Global Burden of Iodine Deficiency: Insights and Projections to 2050 Using XGBoost and SHAP**

### Supplemental Figure 8. SHAP summary plot and dependence plots for features in the original XGBoost model predicting the iodine deficiency prevalence rate in Democratic Republic of the Congo


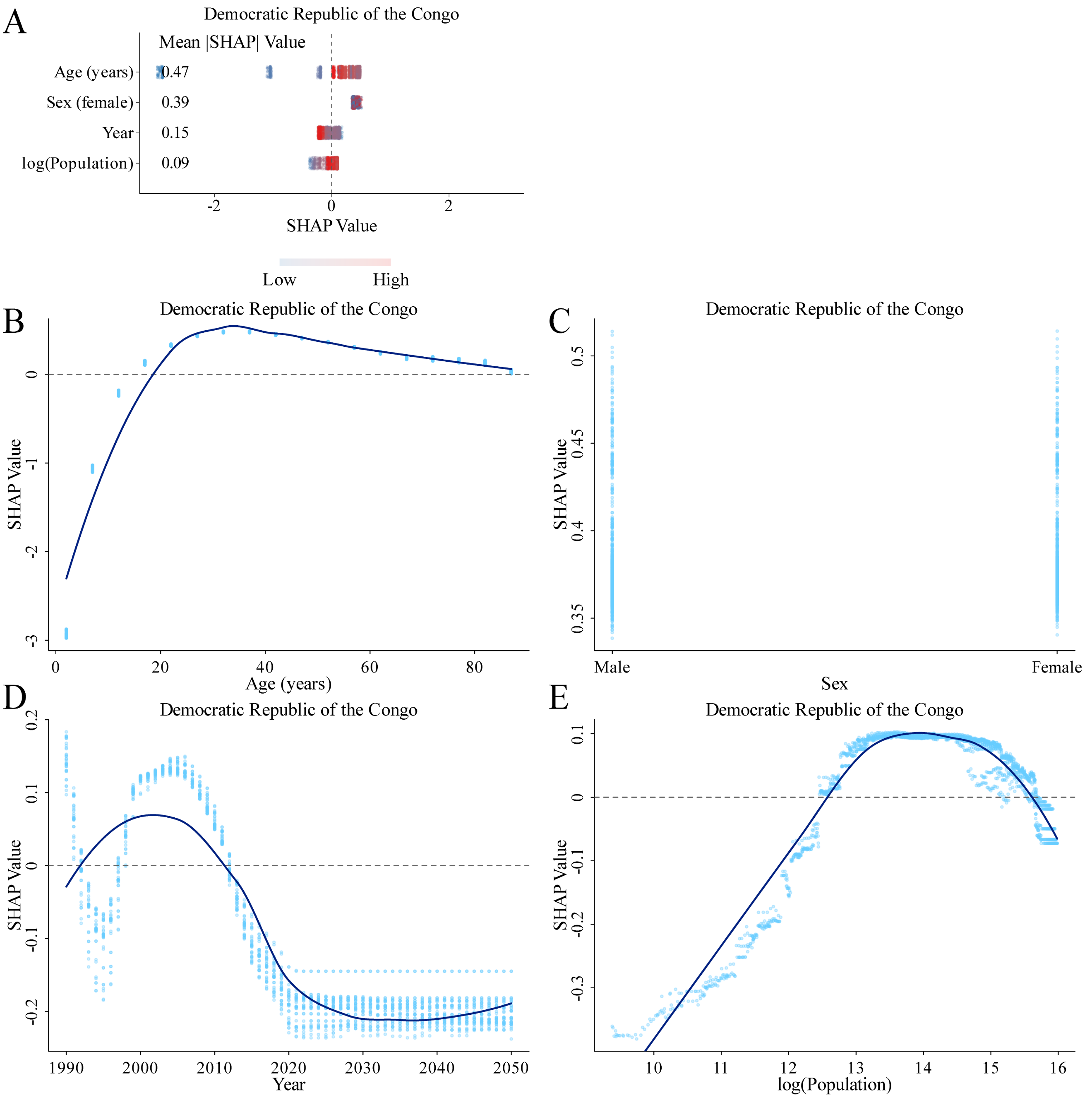


SHAP summary plot (A); Dependence plot of age (B), sex (C), year (D), and log(population) (E).

**Abbreviations:**

SHAP, SHapley Additive exPlanations

**Liang Dan, Wang Li et al. Global Burden of Iodine Deficiency: Insights and Projections to 2050 Using XGBoost and SHAP**

### Supplemental Figure 9. SHAP summary plot and dependence plots for features in the original XGBoost model predicting the iodine deficiency prevalence rate in Djibouti


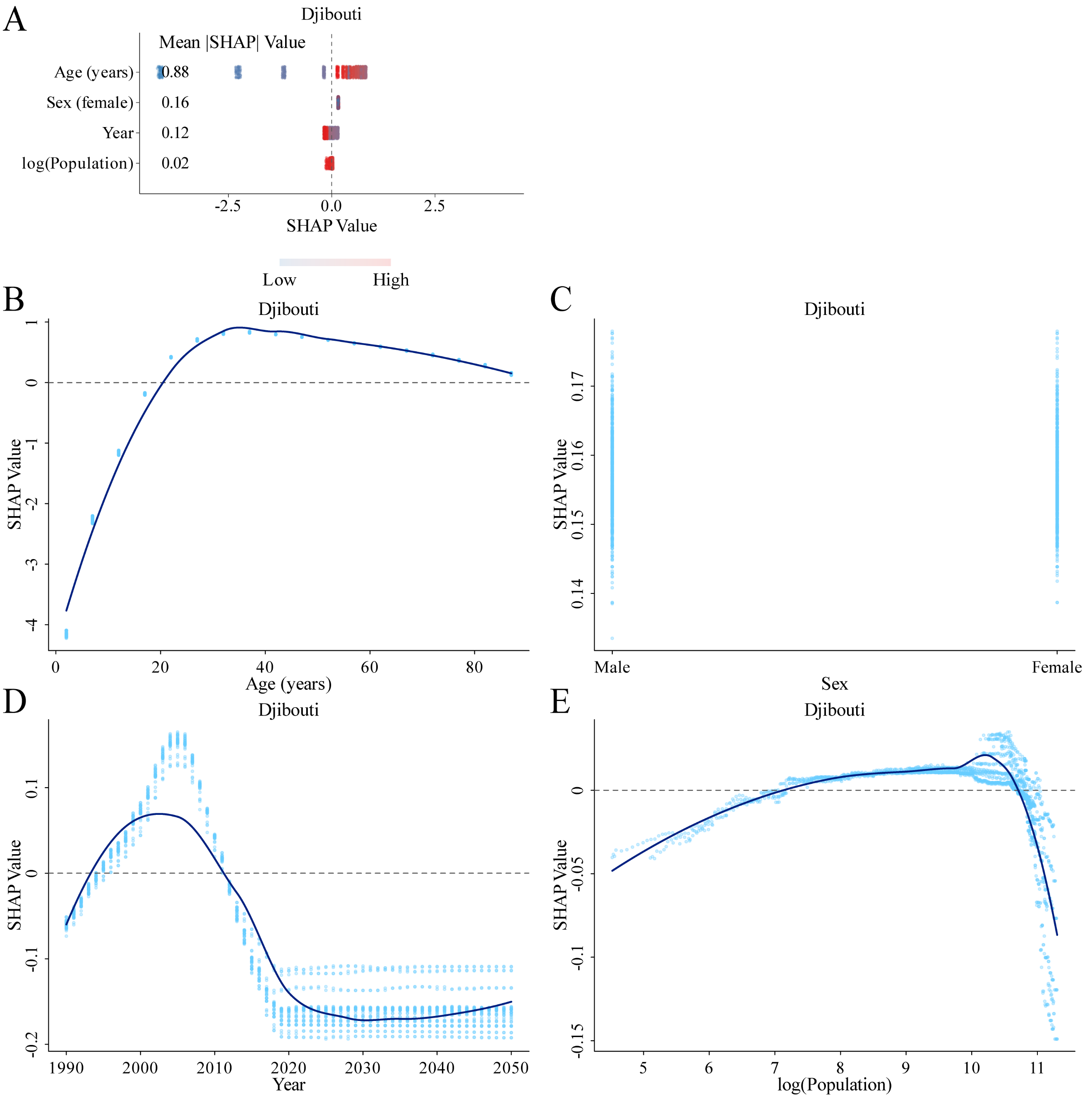


SHAP summary plot (A); Dependence plot of age (B), sex (C), year (D), and log(population) (E).

**Abbreviations:**

SHAP, SHapley Additive exPlanations

**Liang Dan, Wang Li et al. Global Burden of Iodine Deficiency: Insights and Projections to 2050 Using XGBoost and SHAP**

### Supplemental Figure 10. SHAP summary plot and dependence plots for features in the original XGBoost model predicting the iodine deficiency DALYs rate in Somalia


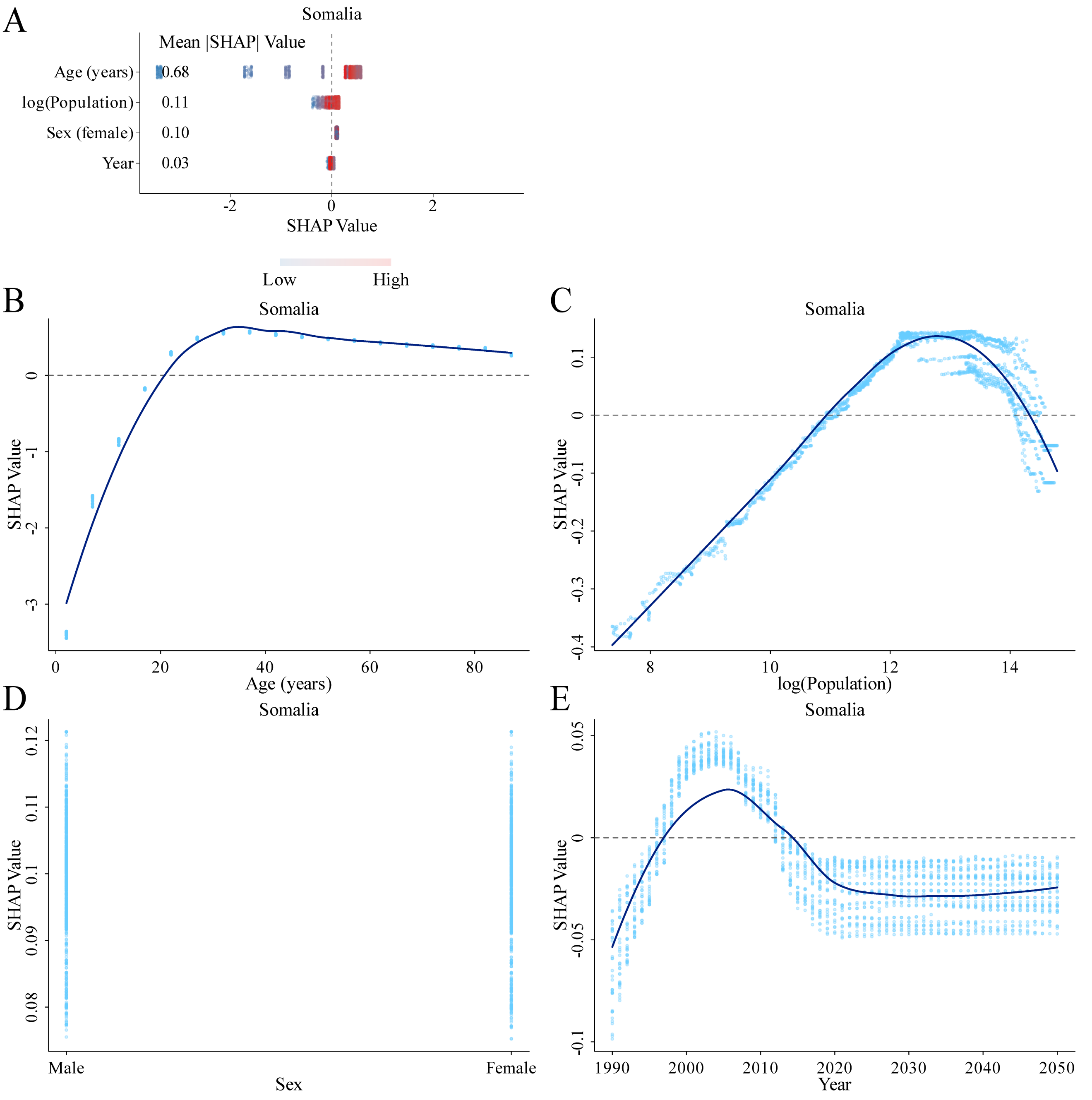


SHAP summary plot (A); Dependence plot of age (B), log(population) (C), sex (D), and year (E).

**Abbreviations:**

SHAP, SHapley Additive exPlanations

**Liang Dan, Wang Li et al. Global Burden of Iodine Deficiency: Insights and Projections to 2050 Using XGBoost and SHAP**

### Supplemental Figure 11. SHAP summary plot and dependence plots for features in the original XGBoost model predicting the iodine deficiency DALYs rate in Democratic Republic of the Congo


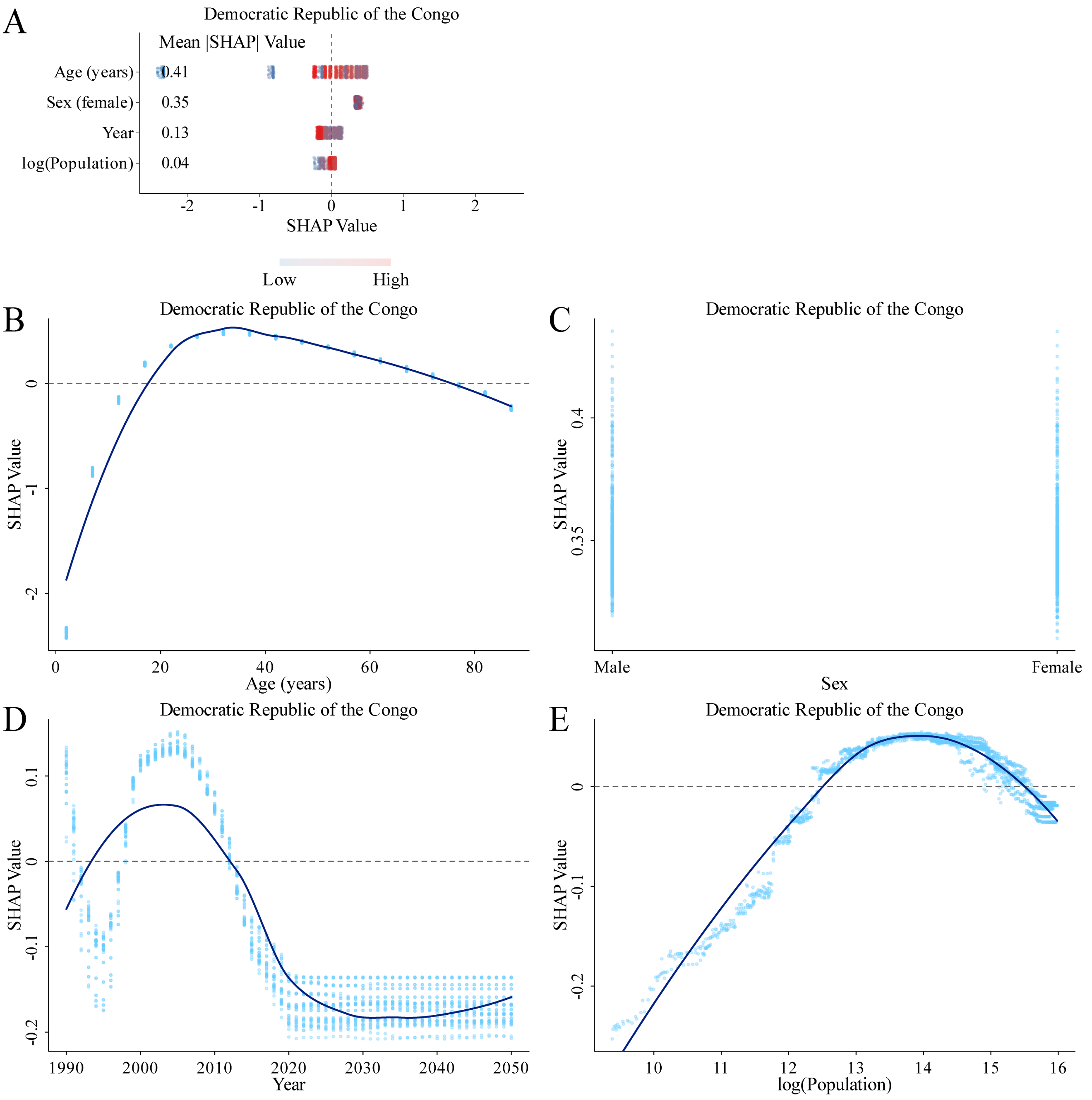


SHAP summary plot (A); Dependence plot of age (B), sex (C), year (D), and log(population) (E).

**Abbreviations:**

SHAP, SHapley Additive exPlanations

**Liang Dan, Wang Li et al. Global Burden of Iodine Deficiency: Insights and Projections to 2050 Using XGBoost and SHAP**

### Supplemental Figure 12. SHAP summary plot and dependence plots for features in the original XGBoost model predicting the iodine deficiency DALYs rate in Djibouti


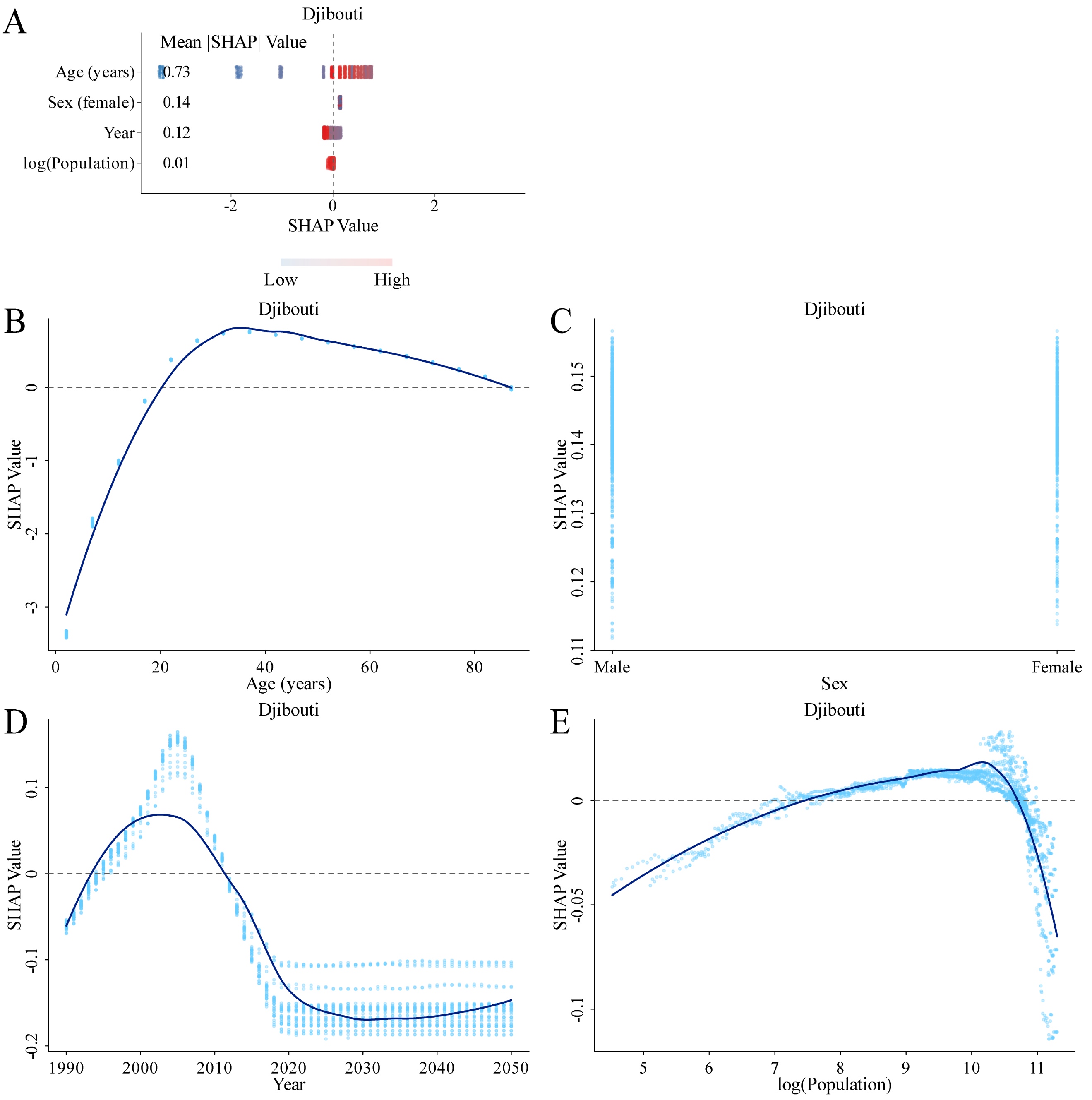


SHAP summary plot (A); Dependence plot of age (B), sex (C), year (D), and log(population) (E).

**Abbreviations:**

SHAP, SHapley Additive exPlanations

**Liang Dan, Wang Li et al. Global Burden of Iodine Deficiency: Insights and Projections to 2050 Using XGBoost and SHAP**

### Supplemental Figure 13. SHAP dependence plots for features in the iodized salt coverage-based XGBoost model predicting the iodine deficiency incidence, prevalence, and DALYs rate in Congo, Democratic Republic of the Congo, and Ethiopia


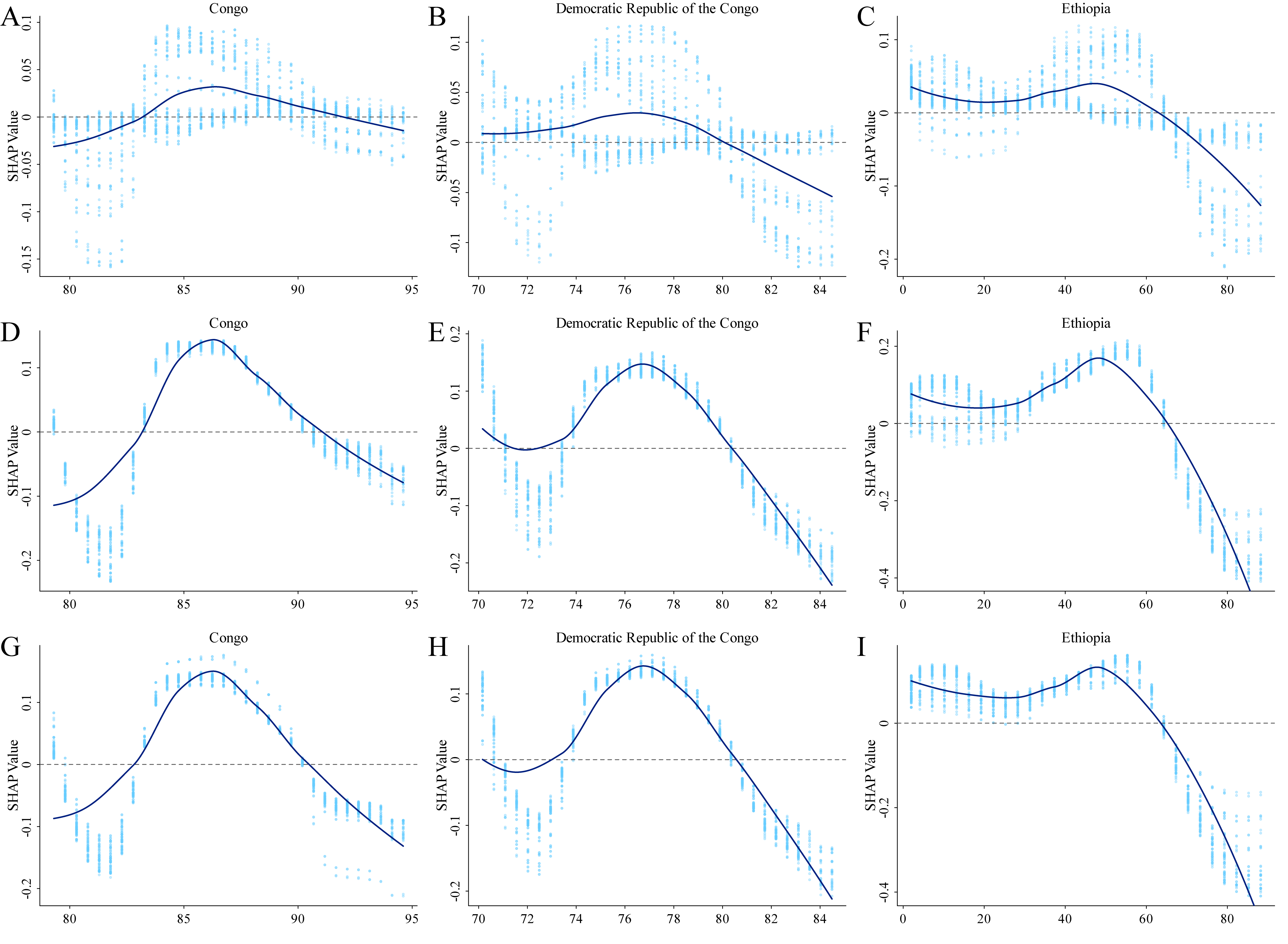


SHAP dependence plot of iodized salt coverage for ID incidence in Congo (A), Democratic Republic of the Congo (B), and Ethiopia (C).

SHAP dependence plot of iodized salt coverage for ID prevalence in Congo (D), Democratic Republic of the Congo (E), and Ethiopia (F).

SHAP dependence plot of iodized salt coverage for ID DALYs in Congo (G), Democratic Republic of the Congo (H), and Ethiopia (I).

**Abbreviations:**

ID, iodine deficiency

SHAP, SHapley Additive exPlanations

DALYs, disability-adjusted life years

**Liang Dan, Wang Li et al. Global Burden of Iodine Deficiency: Insights and Projections to 2050 Using XGBoost and SHAP**

### Supplemental Figure 14. Weighted correlation analysis between the iodized salt coverage and ID ASIR, ASPR, and ASDR at national levels in 1990, 2021, with incident case, prevalent case, and DALYs as weights


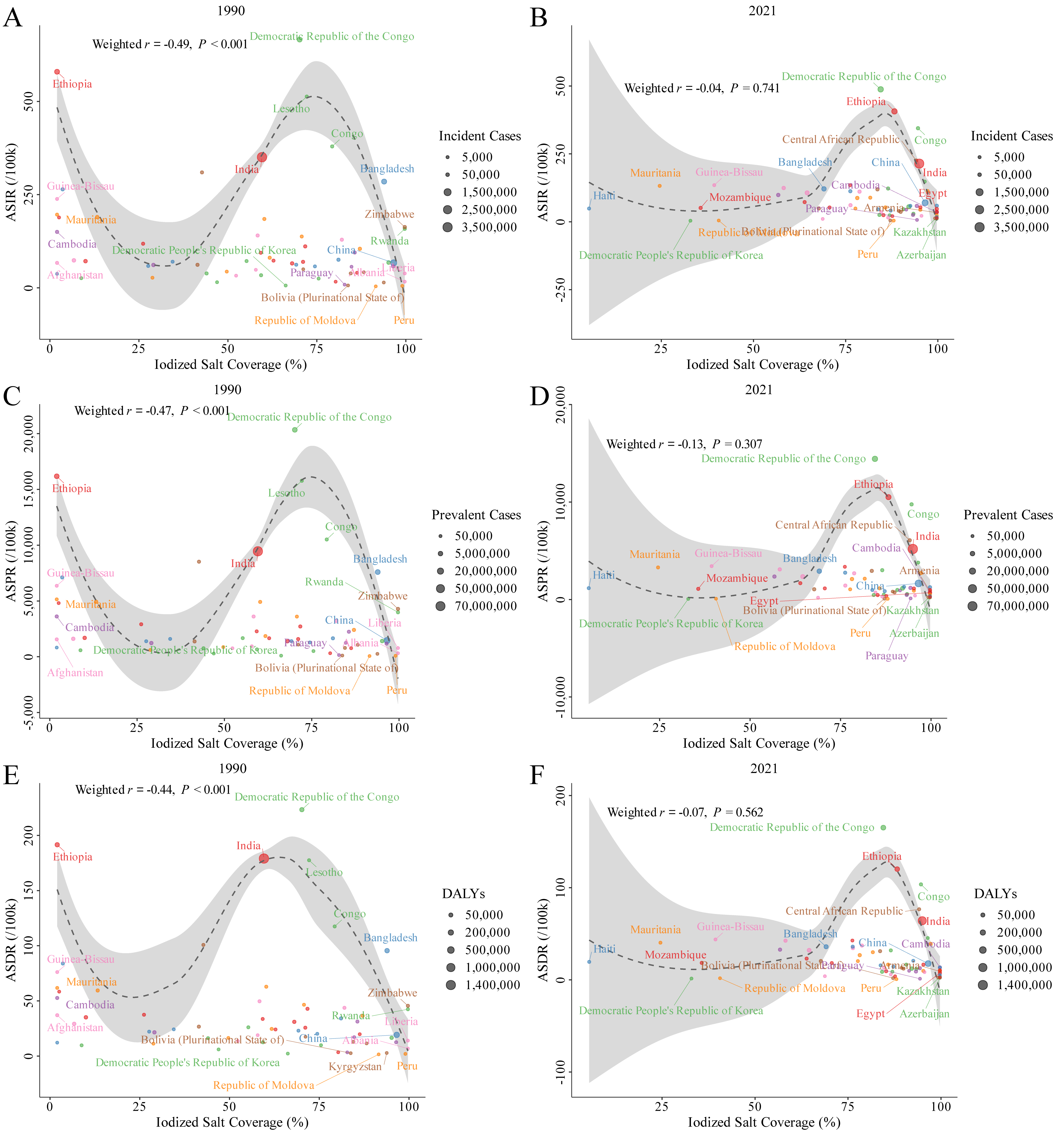


ASIR in 1990 (A) and 2021 (B); ASPR in 1990 (C) and 2021 (D); ASDR in 1990 (E) and 2021 (F); Abbreviations: ID, iodine deficiency; ASR, age-standardized rate; ASIR, age-standardized incidence rate; ASPR, age-standardized prevalence rate; ASDR, age-standardized DALYs rate; DALYs, disability-adjusted life years.

**Liang Dan, Wang Li et al. Global Burden of Iodine Deficiency: Insights and Projections to 2050 Using XGBoost and SHAP**

### Supplemental Movie 1. ASIR of ID at the national level from 1990 to 2050

**Abbreviations:**

ID, iodine deficiency

ASIR, age-standardized incidence rate

**Liang Dan, Wang Li et al. Global Burden of Iodine Deficiency: Insights and Projections to 2050 Using XGBoost and SHAP**

### Supplemental Movie 2. ASPR of ID at the national level from 1990 to 2050

**Abbreviations:**

ID, iodine deficiency

ASPR, age-standardized prevalence rate

**Liang Dan, Wang Li et al. Global Burden of Iodine Deficiency: Insights and Projections to 2050 Using XGBoost and SHAP**

### Supplemental Movie 3. ASDR of ID at the national level from 1990 to 2050

**Abbreviations:**

ID, iodine deficiency

ASDR, age-standardized DALYs rate

DALYs, disability-adjusted life years
